# Studying the structure–property relationship in Zr-BDI MOF for noble-metal-free selective photocatalytic CO_2_ conversion to CO

**DOI:** 10.1039/d6ra07959h

**Published:** 2026-11-02

**Authors:** Akhil Chandran P., Tushar S. Verma, Shreya Sarkar, Ankur Chanda, Ayalew H. Assen, Amit Nag, Ravichandar Babarao, Sounak Roy, Himanshu Aggarwal

**Affiliations:** a Department of Chemistry, Birla Institute of Technology and Science Hyderabad Campus Hyderabad 500078 India himanshu.aggarwal@hyderabad.bits-pilani.ac.in; b Materials Centre for Sustainable Energy & Environment (McSEE), Birla Institute of Technology and Science Hyderabad Campus Hyderabad 500078 India; c School of Science, RMIT University GPO Box 2476 Melbourne Victoria 3000 Australia; d College of Chemical Sciences and Engineering (CCSE), Applied Chemistry and Engineering Research Centre of Excellence (ACER CoE), Mohammed VI Polytechnic University (UM6P) Ben Guerir 43150 Morocco

## Abstract

Photocatalytic conversion of CO_2_ to value-added products by metal–organic framework (MOF)-based catalysts often requires the introduction of secondary metal sites or the anchoring of noble-metal-based photosensitizers, leading to complex designs and increased costs. In this work, we focus on the structure–property relationship of MOFs, emphasizing the band gap and conduction band position of the MOF catalyst. Here, we use a light-sensitive linker [*N*,*N*′-di(3,5-dicarboxyphenyl)benzene tetracarboxylic diimide] to design a robust, intrinsically photo-redox-active zirconium MOF. This Zr-MOF with scu topology has a band gap of 2.84 eV and a conduction band position of −0.64 V *vs.* NHE, which is well positioned for the conversion of CO_2_ to CO without the addition of noble metal atoms or photosensitizers. Interestingly, anion radical generation by the linker under light irradiation supports linker-to-cluster charge transfer and simultaneously enables excellent conversion of CO_2_ to CO and formate, with yields of 1083.2 µmol g^−1^ and 265.62 µmol g^−1^, respectively, and a CO selectivity of 72.12%, surpassing most noble-metal-free Zr-MOF benchmarks. *Operando* Raman spectroscopy has been used to identify key intermediates, including *CO_2_^−^, *COOH, *CO, formyl, and O-bound methoxy species. DFT studies corroborate the experimental findings, showing that the free energy profile indicates a lower barrier for *CO than for formyl formation. This linker-first strategy offers an easy, cost-effective, and interesting approach in which judicious choice of the photo-redox linker can modulate the band-edge alignment and microenvironment of the MOF, enabling selective CO_2_ to CO photocatalysis without precious metals.

## Abstract 2

Here, we report a Zr-based MOF for noble-metal-free photocatalytic conversion of CO_2_ to CO with more than 70% selectivity.

## Graphical abstract

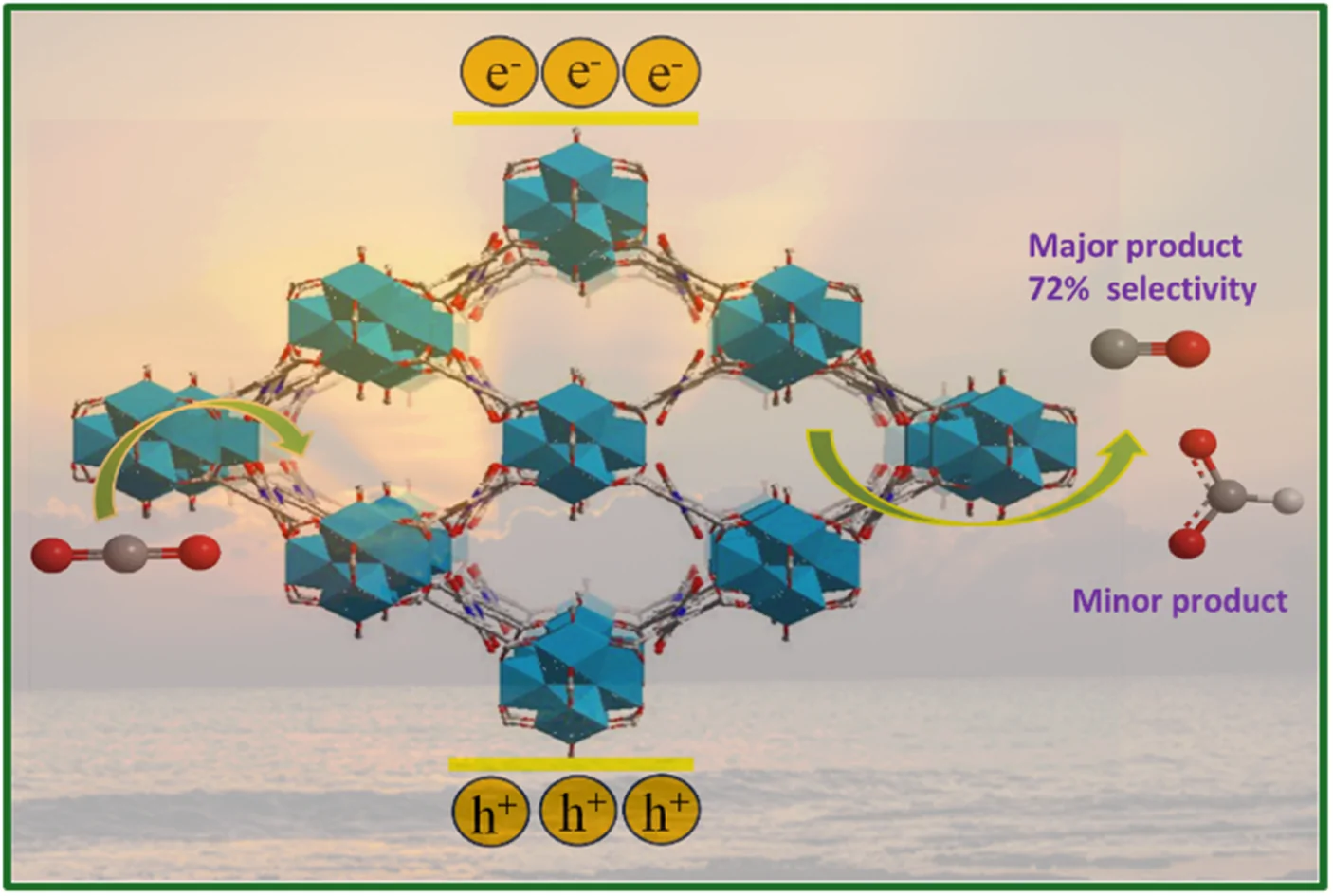

## Introduction

The rapid pace of industrialization and technological advancement has driven a substantial rise in greenhouse gas emissions, with carbon dioxide (CO_2_) being a primary contributor to global warming and environmental degradation.^1–6^ Carbon capture and utilization (CCU) strategies that transform CO_2_ into value-added products offer a complementary pathway to geological storage, enabling circular carbon approaches such as power-to-chemicals and power-to-fuels that integrate with intermittent renewable electricity.^7,8^ In principle, CO_2_ can be upgraded thermally, electrochemically, or photocatalytically; however, its exceptional thermodynamic stability and linear, low polarizability electronic structure make activation difficult. The strong C=O bonds, with a bond energy of approximately 750 kJ mol^−1^, combined with the need for multi-electron, proton-coupled electron transfer steps, result in sluggish kinetics and substantial overpotentials. As a result, effective catalysts must not only harvest energy efficiently but also provide active sites and microenvironments that lower activation barriers, stabilize key intermediates, and improve product selectivity.^3,9,10^

The photocatalytic reduction of CO_2_ can produce a range of products, from C1 species such as CO, CH_4_, CH_3_OH, or HCOOH to higher-energy-density C2 products like CH_3_COOH, C_2_H_4_, or CH_3_CH_2_OH.^11^ Achieving high selectivity in photocatalytic CO_2_ reduction is challenging, and the product control requires careful band edge alignment and local microenvironment engineering.^12,13^ For selective CO formation, the conduction band (CB) should be positioned more negative than *E*_0_ (CO_2_/CO) ≈ −0.11 V *vs.* RHE to drive the two-electron reduction, while not providing excessive driving force toward the competing hydrogen evolution reaction. Simultaneously, the valence band (VB) should be more positive than the water oxidation potential (+1.23 V *vs.* RHE) to enable efficient hole utilization and suppress electron–hole recombination.^14^ Noble metal-based semiconductors offer high efficiency for CO_2_ conversion but are prohibitively costly or scarce for large-scale applications.^9,10,15,16^

Metal–organic frameworks (MOFs) are fascinating photocatalyst platforms because their crystalline, modular architectures enable independent tuning of pore structure, active-site chemistry, and electronic properties. High surface areas and ordered porosity facilitate CO_2_ uptake and mass transport, while the periodic arrangement of organic linkers and inorganic nodes enables precise placement of functional groups near catalytic centers.^7,17,18^ In many MOF-based systems, photocatalysis proceeds through linker to cluster charge transfer (LCCT), where π-conjugated linkers absorb visible light, generate excitons, and inject electrons into oxocluster nodes that mediate substrate activation and redox turnover.^12,18–24^

Drawing on existing literature, we critically examined the relationships among structure, electronic properties, and photocatalytic performance in reported MOF-based CO_2_ reduction systems. Robust zirconium MOFs such as UiO-66 and MOF-808, although structurally stable, possess wide band gaps and unfavorable band-edge positions, necessitating post-synthetic strategies such as solvent-assisted ligand exchange or the incorporation of noble-metal-based photosensitizers to enable visible-light activity and efficient charge transfer.^12,14,17,25,26^ While these approaches have demonstrated improved photocatalytic performance, they inherently increase material complexity and cost. Recent efforts, therefore, focus on the rational design of intrinsically photoactive MOFs that meet the band-gap and conduction-band criteria required for CO_2_ reduction without relying on external photosensitizers.^18,27^ The planarity and rigidity of diimide cores support strong light absorption and can facilitate charge transport *via* redox hopping, whereas high framework connectivity mitigates linker leaching and photobleaching under operation.^28–30^ Together, these features make *N*,*N*′-di(3,5-dicarboxyphenyl)benzene tetracarboxylic diimide (BDI) based Zr-MOFs well-suited for integrating light harvesting, charge transfer, and catalytic site engineering for the selective conversion of CO_2_ to CO.

Under visible-light irradiation in aqueous media, the MOF produces CO as the major product and formate and methane as minor coproducts, indicating selective channeling of photogenerated carriers and suppression of HER. The source of improved CO selectivity is further investigated using *in situ* Raman spectroscopy and complementary computational studies, which provide a mechanistic understanding of the interplay among ligand design, band alignment, and photocatalytic activity.

## Experimental methods

### Materials

Pyromellitic dianhydride (benzene 1,2,4,5-tetracarboxylic dianhydride, 97%) was ordered from Sigma Aldrich. 5-Aminoisophthalic acid (99%) and ZrOCl_2_·8H_2_O (98%) was purchased from SRL Chemicals. Solvents such as DMF and Chlorobenzene were purchased from SRL Chemicals, and the modulator formic acid (97%) was ordered from Alfa Aesar.

### Ligand synthesis

Ligand synthesis was carried out from the reported procedure. In a 100 ml round bottom flask, 5-aminoisophthalic acid (5.73 mmol) and pyromellitic dianhydride (2.29 mmol) were mixed with 15 ml of glacial acetic acid. The mixture was stirred to ensure homogeneity before being refluxed at 130 °C for 24 h. The resulting whitish grey precipitate (*N*,*N*′-di-(phenyl-3, 5-dicarboxylic acid)-benzene tetracarboxylic acid diimide) was washed with water and then methanol. Recrystallization was done in DMF.

### MOF synthesis

ZrOCl_2_·8H_2_O (0.067 mmol) and *N*,*N*′-di-(phenyl-3, 5-dicarboxylic acid)-benzene tetracarboxylic acid diimide (0.017 mmol) were mixed in a 20 ml scintillation vial and dissolved in 2.5 ml DMF through sonication. 1 ml of chlorobenzene and 2 ml of formic acid were added, and the mixture was sealed with a cap. Vials were kept in 130 °C for 48 h resulting in light greenish crystals. Crystals were washed with DMF (15 ml × 3) before use.

### Characterization

Ligand structure was determined using Nuclear Magnetic Resonance (NMR). Bruker AVANCE NEO 400 MHz FT-NMR Spectrometer was used for ^1^H NMR and ^13^C NMR analysis. The crystallinity and bulk purity of the sample were determined using a Rigaku Ultima IV X-ray diffractometer with Cu Kα (*λ* = 1.54 A°) source (40 kV, 40 mA). The measurement was performed from 3° to 40° at a scan rate of 2° min^−1^ and a step width of 0.01. A nitrogen adsorption isotherm was collected to determine the surface area and pore parameters of the prepared sample using an Anton Paar Autosorb instrument. Before surface area measurement the Zr BDI MOF was exchanged with acetone (washed twice a day with fresh acetone) for three days and activated at a temperature of 100 °C for 4 h. Also, the sample was checked for its capacity for different gas adsorption using a high-pressure adsorption instrument (Anton Paar). The sample was heated in an inert atmosphere (N_2_) at a heating rate of 10 °C per minute from room temperature to 900 °C in a SHIMADZU DTG-60 instrument to understand the thermal behaviour. The surface morphology of the crystals was explored using the Scanning Electron Microscopy FEI Apreo LoVac instrument. Also, the percentage of the elements were calculated using EDS mapping. Another method used for elemental analysis and binding energy calculations to identify the bonds present was X-ray photoelectron Spectroscopy. A Thermo Scientific K-α instrument with Al Kα micro-focused monochromator of energy 1486.8 eV and work function (*φ*_SP_) 4.244 eV were used for excitation. The elements present and the type of bonds present in the MOF were detected from the binding energy. Avantage software was used for spectra deconvolution with a C 1s peak at 284.85 eV as the reference. The valence band position is also determined from XPS (*E*_eV_). Using the equation below, it is converted to the NHE scale (*E*_NHE_), which agreed with other band property measurements.*E*_NHE_ = *E*_eV_ + *φ*_SP_ − 4.44

Valence band (*E*_VB_) position was calculated by the equation.*E*_VB_ = 21.2 − (*E*_SECO_ + *E*_HOMO_)*E*_SECO_ – secondary electron cut-off energy. *E*_HOMO_ – binding energy of the highest occupied molecular orbital.

For checking bandgap and photoabsorption properties, the solid-state absorption and emission spectra were recorded using a Jasco V-670 UV-vis spectrometer and a Jasco FP-6300 spectrofluorometer, respectively. A Bruker Magnettech ESR 5000 instrument was used for recording the EPR signal. The sample was dispersed in acetonitrile and filled in the capillary tube. EPR spectra were collected in the dark condition as well as after exposing the sample to UV radiation.

The *g*-factor was found using the equation given below.*g* = *hν*/*µ*_B_*B**h* – Planck's constant. *ν* – Microwave radiation frequency. *µ*_B_ – Bohr magneton. *B* – Magnetic field.

### Photocatalytic experiment

The as-synthesized samples were washed thoroughly in DMF to remove undissolved residues and then exchanged with acetone for 3 days. Afterward, it was activated for 4 h at 100 °C to remove the solvents. 50 mg of the catalyst was dispersed in 25 ml of the medium. The reaction medium contained 24 ml water and 1 ml triethanol amine (TEOA). A high-pressure mercury vapor lamp with a 250 W power source is used as the excitation light source. Argon gas was passed at a rate of 1 cm^3^ min^−1^ for 20 min to remove dissolved oxygen and to create an inert atmosphere. Later, CO_2_ (99.9%) was passed at a rate of 1 cm^3^ min^−1^, and the reaction was carried out at ambient temperature and pressure conditions. 1 ml solvent medium was collected each hour, centrifuged, and the fresh residues were collected. 600 µl parent solution, 100 µl D_2_O, and 100 µl *P*-nitrophenol (35.5 mmol) were mixed, and NMR analysis was carried out. The CO_2_ reduction product is identified by NMR and quantified relative to the standard *P*-nitrophenol (PNP). The analysis of gaseous products was carried out at similar intervals using an Agilent 8890 gas chromatography equipment. The product was quantified using a standard calibration graph obtained from known concentrations of CO. The experiment was performed multiple times, and the standard deviation of the values was plotted as error bars.

### Crystal structure determination

The structure of Zr-BDI-scu-MOF was constructed using Materials Studio by modifying the previously reported Zr-NDI-scu-MOF.^31^ This modification involved substituting the NDI linker with the BDI linker while preserving the zirconium hexanuclear cluster coordinated to eight carboxylate groups from the linker. The replacement process utilized the “Sketch Atom” tool in Materials Studio after manually removing NDI linker atoms that did not align with the corresponding positions in the BDI linker. Since formic acid was used as a modulating agent in the synthesis, the remaining four coordination sites of the Zr hexanuclear cluster were completed by formate groups. Before substituting the linker atoms, bound and unbound solvent molecules in the Zr-NDI-scu-MOF structure were manually removed. The Zr-BDI-scu-MOF structure was then optimized using Materials Studio 2017. Geometry optimization was conducted using the Smart algorithm in the Forcite module, with a maximum of 1000 iterations. Energy minimization was performed with a convergence tolerance of 10–4 kcal mol^−1^. The convergence thresholds for geometry optimization were set to 10–6 Å for displacement, 10–6 kcal mol^−1^ for energy, and 10–6 kcal mol^−1^ Å for force. The framework's cell parameters were allowed to relax during the optimization process.

### Electrochemical measurement

A pseudo-airtight cell was used to construct the three-electrode setup dipped in 0.2 M Na_2_SO_4_ (pH-7). A 3 mm diameter Glassy carbon electrode (GCE), an Ag/AgCl electrode, and a platinum rod electrode were used as the working electrode, reference electrode, and counter electrode, respectively. 5 mg of the activated MOF was homogeneously dispersed in 1 ml of methanol and 10 µl of Nafion *via* sonication. 20 µl of this mixture was left to evaporate on the GCE, forming a uniform layer of catalyst. The conducting property of the MOF was evaluated using electrochemical impedance measurements over the 1 Hz to 1 MHz frequency range. Also, the position of the conduction band was checked through the Mott–Schottky plot at different frequencies. All studies were conducted at room temperature using an Origaflex Origa 5000 potentiostat.

### *In situ* Raman spectroscopy

The Raman measurements were carried out using a UniDRON-A micro-Raman mapping spectrometer. A total of 5 mg of the catalyst was sonicated in 1 ml of methanol, and 20 µl of the resulting dispersed solution was drop-cast onto a 1 × 1 cm^2^ silicon wafer. The wafer was then activated at 80 °C to allow for complete drying. Raman spectra were recorded using a continuous-wave diode-pumped solid-state laser (*λ* = 532 nm, ∼10 mW power). To collect the backscattered Raman light during catalysis, the sample was focused using a 50× air immersion objective lens with a 0.55 numerical aperture. Before collecting the Raman spectra of the samples, the spectrograph was calibrated using a standard silicon wafer at 520 cm^−1^. Subsequently, the sample was immersed in a Petri dish containing a highly saturated aqueous CO_2_ solution, and the laser was irradiated to collect spectra.

## Results and discussion

The BDI ligand was synthesized according to a reported procedure,^30^ and its structure was confirmed by ^1^H NMR (Fig. S1) and ^13^C NMR (Fig. S2). The BDI ligand was chosen for its photo-redox activity, which will further enhance the photocatalytic activity. The redox nature of the BDI ligand was checked in an electrolyte solution (0.2 M Na_2_SO_4_, pH = 7), which showed a redox peak at a potential of −0.67 V *vs.* Ag/AgCl (Fig. S3). The EPR spectra of the ligand under light conditions displayed a high-intensity peak at *g* = 2.003, further confirming anion radical formation upon light irradiation (Fig. S4). Based on the above observations, a possible mechanism for radical formation in the ligand during light irradiation is proposed through a redox-active route (Fig. S5). The Zr-BDI MOF crystals were synthesized solvothermally using a modulator approach. Single-crystal X-ray diffraction (SCXRD) data collection was not possible because the as-synthesized crystals obtained were too small. To gain structural insight, the framework was then modeled in Materials Studio using a closely related MOF structure as a reference, *i.e.*, Zr-NDI-scu-MOF.^31^ The simulated Zr-BDI-scu-MOF structure (Fig. 1) was then used to calculate the corresponding XRD pattern. Notably, the calculated diffraction pattern aligns very well with the experimental PXRD data (Fig. 2a), confirming that the simulated model accurately represents the actual crystalline phase. Despite the lack of single-crystal data, this agreement provides strong evidence that the synthesized Zr-BDI MOF has a 4,8-c structure with a scu topology, in which each BDI linker is bonded to four Zr-hexanuclear clusters, and each Zr-hexanuclear cluster is connected to eight BDI ligands (Fig. 1). The matching of PXRD of the simulated structure with the experimental PXRD (Fig. 2a) further confirms the bulk phase purity of the synthesized MOF.

**Fig. 1:**
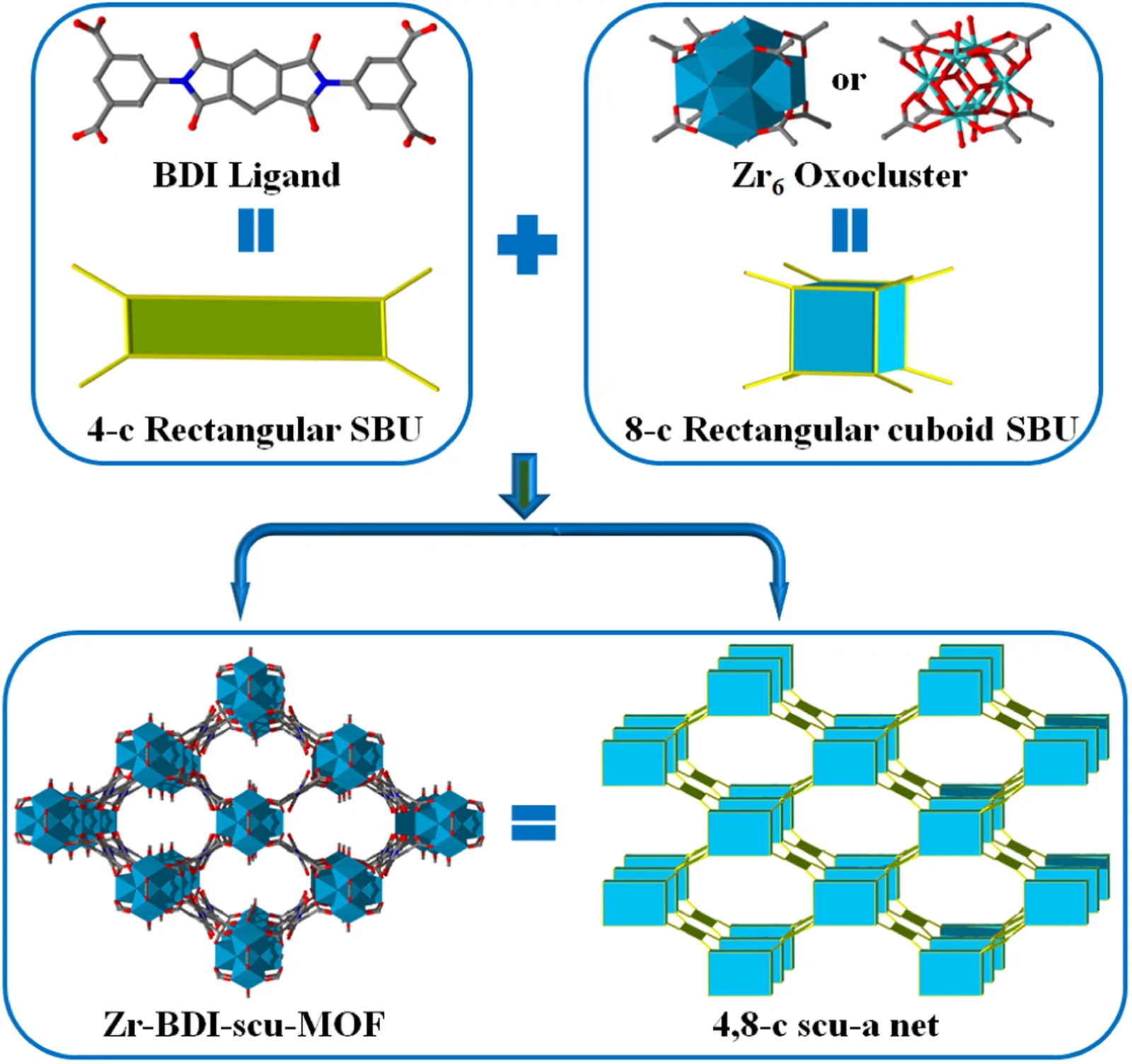
Schematic showing the construction of Zr-BDI-scu-MOF structure from the tetratopic BDI ligand and Zr hexanuclear cluster along with the framework topology representation of 4,8-c augmented scu-a net.

**Fig. 2:**
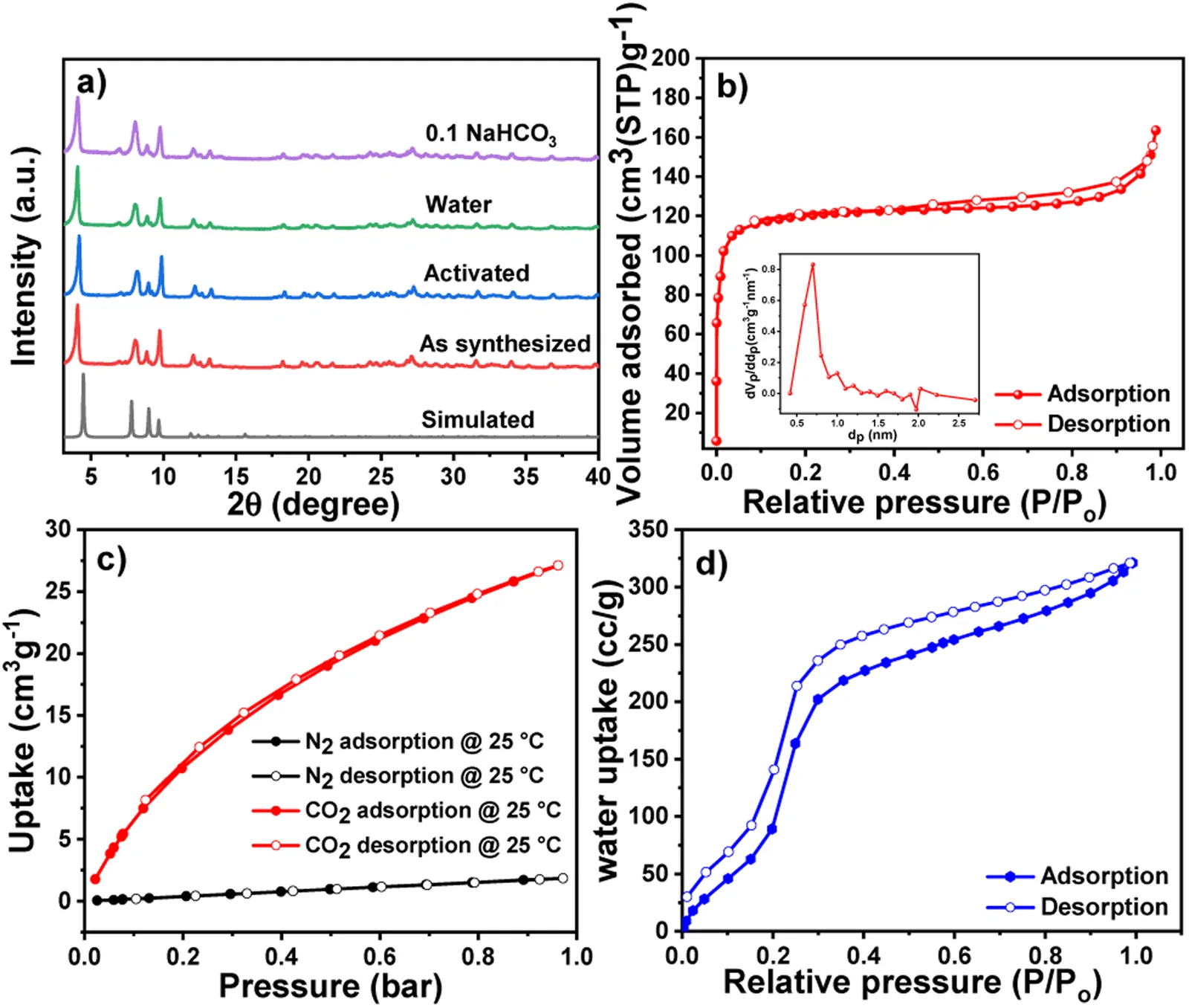
(a) PXRD comparison of the as-synthesized MOF with the simulated pattern along with stability in different solvent media, (b) N_2_ sorption isotherm collected at 77 K and pore distribution curve (inset). (c) Comparison of N_2_ and CO_2_ sorption at STP. (d) Water sorption isotherm at 298 K.

The Brunauer–Emmett–Teller (BET) surface area analysis was performed after collecting the N_2_ adsorption isotherm at 77 K up to a relative pressure of 1.0. The N_2_ isotherm (Fig. 2b) exhibits a type I profile, indicating a predominantly microporous structure. A small hysteresis loop appears at higher relative pressures, which may result not from intrinsic mesoporosity but either from slight framework flexibility or from interparticle condensation and textural voids caused by the packing of the very small crystallites. BET analysis conducted within the appropriate linear range yields a specific surface area of 524 m^2^ g^−1^, while the total pore volume at *P*/*P*_0_ ≈ 0.95 is 0.26 cm^3^ g^−1^ and the average pore diameter is 6.3 Å. Pore diameter and pore volume values of approximately 6.9 × 7.7 Å and 0.47 cm^3^ g^−1^, respectively, were calculated using Materials Studio, further confirming the accuracy of the simulated structure. The MOF does not exhibit the optimal pore volume compared to the calculated value, likely due to the inherent flexibility of the scu framework, which decreases pore accessibility under measurement conditions. To investigate whether mesopores are present at a different site, a BJH plot was generated from the N_2_ isotherm analysis; however, no traces of mesopores were found, confirming pure micropore behavior (Fig. S6). The CO_2_ adsorption isotherm recorded at 1 bar and 298 K showed an uptake of nearly 27 cm^3^ g^−1^, while the adsorption of N_2_ at room temperature was negligible (Fig. 2c). The isosteric enthalpy of adsorption for CO_2_ was determined by recording adsorption isotherms at 283 K and 293 K, which showed a *Q*_st_ value of about −31 kJ mol^−1^ at low loading, confirming a good interaction of the CO_2_ molecules with the MOF framework (Fig. S7). High-pressure gas adsorption experiments were conducted using H_2_, N_2_, and CH_4_, with the MOF sample exhibiting uptakes of 26.20 cm^3^ g^−1^, 54.21 cm^3^ g^−1^, and 87.86 cm^3^ g^−1^, respectively (Fig. S8).

The MOF also exhibited good stability upon activation and exposure to humid conditions (for more than a week), basic conditions (for 24 hours), and acidic conditions (formic acid for more than a week), due to the presence of high-valent Zr nodes. Similarly, the framework integrity remained unaffected in the presence of various organic solvents (Fig. S9). MOF also showed a high water uptake capacity of ∼325 cm^3^ g^−1^ with small hysteresis. Interestingly, nearly 200 cm^3^ g^−1^ of water is adsorbed at a low *P*/*P*_0_ of 0.3, which can be attributed to the strong capillary effect associated with the filling of the MOF pores (Fig. 2d).

The morphology of the crystals was investigated using Scanning Electron Microscopy (SEM). SEM images revealed that rod-shaped Zr MOF microcrystals were formed (Fig. S10). Furthermore, the elemental composition was determined using energy-dispersive X-ray spectroscopy (EDS). Zirconium, carbon, oxygen, and nitrogen were present at atomic percentages of 4.68%, 68.47%, 25.11%, and 1.74%, respectively, indicating the sample's surface purity (Fig. S11 and Table S1). X-ray photoelectron spectroscopy technique supported the elemental distribution on the surface of the MOF (Fig. 3a). A survey spectrum of the sample revealed the presence of zirconium, carbon, nitrogen, and oxygen, as previously observed by SEM-EDS. The core-level spectra were deconvoluted to identify specific bonds. C 1s spectra revealed peaks at 284.65 eV, 285.30 eV, 286.38 eV, and 288.64 eV, indicating C–C, C–N, C–O, and C=O groups, respectively (Fig. 3b). Zr 3d XPS spectra is split due to spin–orbit coupling, and the binding energies corresponding to 3d_5/2_ and 3d_3/2_ are 182.6, 185.1, respectively, which indicate the existence of Zr in the +4-oxidation state in the secondary building unit (Fig. 3c). Also, from the O 1s spectra, the peaks corresponding to the COOH functional group, Zr–O–Zr bonding in the hexanuclear cluster, and Zr–O–C were identified at 530.17 eV, 533.41 eV, and 531.79 eV, respectively (Fig. 3d).^12,22,32,33^ The thermogravimetric analysis (TGA) revealed the porous nature of the MOF sample, resulting in nearly 19.1% weight loss due to solvent evaporation till 100 °C in the as-synthesized sample. The activated sample showed barely any weight loss till 100 °C (Fig. S12), while the major structural decomposition starts after 400 °C.

**Fig. 3:**
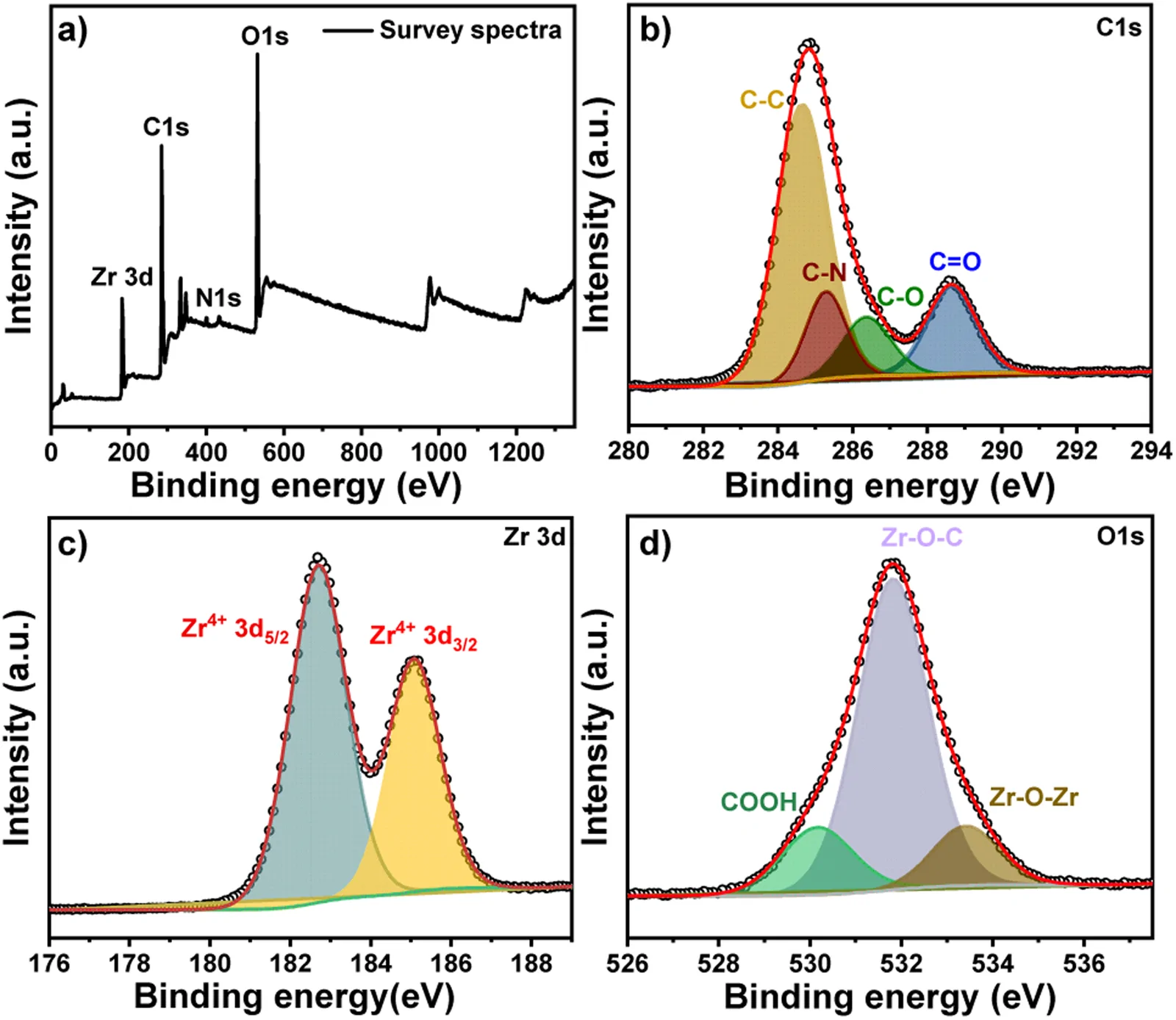
XPS analysis of the MOF showing binding energies and oxidation states. (a) Survey spectra showing elemental distribution in the MOF, (b) C 1s spectra showing different connectivity in the MOF, (c) Zr 3d deconvoluted spectra showing its oxidation state, and (d) O 1s spectra showing bonds associated with O in the MOF.

Before conducting the photocatalytic experiment, the material's electronic properties, including absorbance, band gap, and band positions, must be verified. The BDI ligand showed two distinct absorbance peaks at 332 and 395 nm, which arise from π–π* and n–π* transitions, while the MOF showed the transitions with a shift towards a lower wavelength of 307 and 391 nm, respectively (Fig. 4a). Also, a slight broadening of the peak towards the visible region is also observed in the MOF. This is due to ligand-to-cluster charge transfer (LCCT). For a MOF to act as an effective photocatalyst, the conduction band position of the material must be more negative than that of the standard reduction potential value of CO_2_ to a particular product.^1,3,34,35^ The band gap of the material was determined from the Kubelka–Munk plot [(*K* × *E*)^*n*^ against photon energy; *K* = (1 − *R*)^2^/2*R*] using reflectance spectra. Here ‘*n*’ is the electronic transition function. An *n* value equal to 2 indicates direct allowed transitions, whereas *n* = 1/2 points towards an indirect allowed transition.^36^ A direct bandgap of 2.84 eV and an indirect bandgap of 2.63 eV were obtained for the MOF (Fig. 4b & S13). To assess the thermodynamic feasibility of the targeted photocatalytic application, the position of the conduction band was determined using Mott–Schottky electrochemical measurements at two different frequencies (500 Hz and 1500 Hz). A positive slope of the *C*^−2^*vs. V* proved that the MOF is an n-type semiconductor. The tangent meets the *x*-axis at −0.85 V *vs.* Ag/AgCl, which corresponds to the flat band potential where charge carriers accumulate at the catalyst–electrolyte interface. When converted to the NHE scale, the value is −0.65 V (Fig. 4c).^26^ For n-type semiconductors, the flat band potential is close to the conduction band, where charge carriers can move freely, making it available for the reduction reaction.^37,38^ The photocatalytic experiment was conducted under Water/TEOA condition. The catalyst remained stable in the water medium throughout the reaction time (Fig. 2a). Additionally, it demonstrated stability in the organic medium for over a week.

**Fig. 4:**
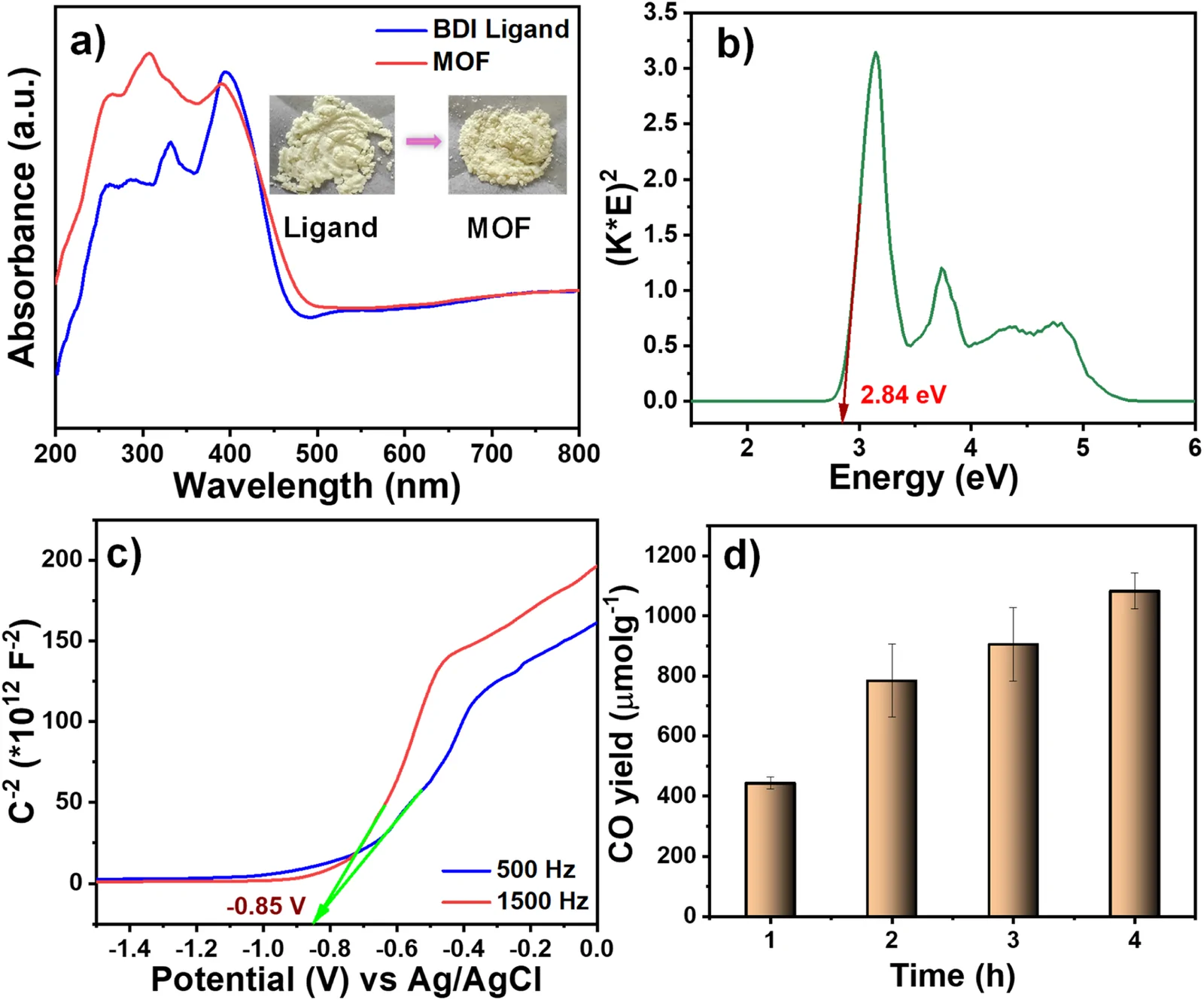
(a) The UV-vis spectra of the MOF and ligand show absorption intensity and peak position changes, (b) The Kubelka–Munk plot determines the band gap of the material, (c) Mott–Schottky plot showing the conduction band position at −0.85 V *vs.* Ag/AgCl (d) CO production during the catalytic reaction.

An initial screening of various media was conducted before the photocatalytic CO_2_ reduction experiment. The reaction was carried out in pure water, under mild base conditions (NaHCO_3_), and in a water/triethanolamine (sacrificial agent) medium. A maximum CO yield was observed when the MOF catalyst in a water/triethanolamine medium was irradiated with visible light (Fig. S14). The light was irradiated after CO_2_ was passed through the medium for 4 hours, and the product was analyzed using NMR. NMR showed a peak at 8.36 ppm corresponding to formate (Fig. S15).^39^

Formate quantification was performed by integrating the area under the curve relative to the *P*-nitrophenol standard (Fig. S15).^2,26^ A maximum of 265.62 µmol g^−1^ of formate was produced in 4 h, resulting in a production rate of 66.40 µmol g^−1^ h^−1^. To ensure that formate is not liberated from the coordinated sites, the catalyst was irradiated with light in water for an extended period, and no trace of formic acid was detected (Fig. S16). Carbon monoxide (CO) was the major product detected in the gaseous product analysis. Additionally, methane was also found as a minor product. An overall yield of 153 µmol g^−1^ of methane was obtained at the end of the reaction. An overall yield of 1083.2 µmol g^−1^ was obtained for CO at the end of the reaction with a production rate of 270.8 µmol g^−1^ h^−1^. Hence, it was concluded that among the gaseous products, the framework showed selectivity for CO as the major product. Notably, this value surpasses most of the Zirconium frameworks that have been reported for CO_2_ reduction without the use of any noble/precious metal (Fig. 4d, Tables S2 and S3). Also, the hydrogen gas was not detected in the GC, which has a minimum detection limit of 100 ppm. Hence, it was concluded that competitive hydrogen production was negligible in this case, thereby enhancing CO production efficiency. An overall carbon-based selectivity of 72.12% for CO, 17.6% for formate, and 10.18% for methane was calculated and reported in the SI. After the first cycle, the catalyst was collected from the reaction medium through centrifugation, dried, and activated at 100 °C. Later, it was further used for the second and third catalytic cycles. Across all cycles, the yield of the major product was similar (Fig. S17). The catalyst showed good stability after the third cycle, too, which was further verified by PXRD. The crystallinity was found to be good, indicating the catalyst's long-term potential (Fig. S18). Also, the XPS spectra of the catalyst were collected at the end of the cycle and were found to be identical to those before the start of the reaction (Fig. S19), further confirming the catalyst's stability after the reaction.

To further elucidate the mechanistic pathway of electron-transport-related reduction at the catalyst surface, solid-state fluorescence spectra were collected. The ligand and MOF sample were excited at 400 nm, and it exhibited a major emission at 469 nm, resulting from the recombination of electron–hole pairs. The fluorescence intensity of the MOF was lower compared to the ligand (Fig. 5a). The lifetime analysis was carried out using a 375 nm laser for excitation. The lifetime of the MOF (0.29 ns) was observed to be higher than that of the ligand (0.11 ns), which indicated the involvement of charge-transfer pathways (LCCT) upon framework formation (Fig. 5b). EPR spectra showed a signal during light irradiation with a *g*-factor of 2.003, corresponding to radical formation and the delocalization of free electrons, which are retained in the MOF, as with the ligand.^34,35^ (Fig. 5c and S4). The prolonged excited-state lifetime of the MOF therefore reflects the generation of longer-lived charge-separated states, in which the ligand-derived radical anions act as transient electron reservoirs. Such stabilized reduced species are highly beneficial for photocatalysis, as they increase the likelihood of interfacial electron transfer to adsorbed reactants, thereby enhancing catalytic efficiency.^40–42^ Due to the production of more electrons during light absorption, it is necessary to verify whether the electrons are provided with a smooth reaction over the catalyst surface. Hence, the impedance spectrum was collected under light-on/off conditions.^12^ In the spectra, the semicircle diameter during the light-on condition was less compared to the light-off condition for the MOF. It signifies that the material offered the electrons low internal resistance to their flow during the photoreaction (Fig. 5d). To further confirm this, the response of the MOF towards the light was recorded by a transient photocurrent experiment under on-off conditions. The stable photocurrent implies better charge separation and transport in the Zr BDI MOF (Fig. S20).

**Fig. 5:**
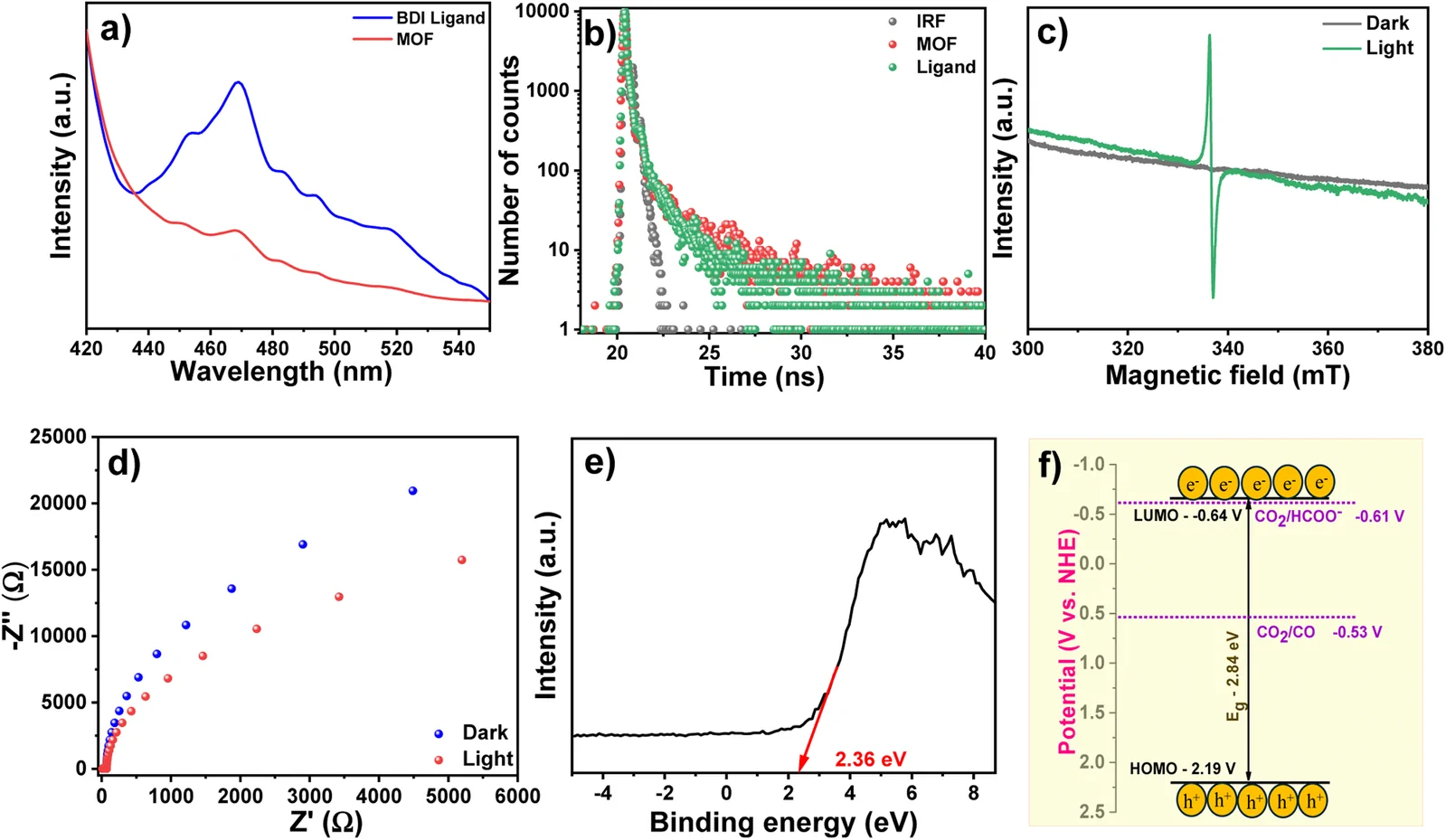
(a) Fluorescence spectra of the MOF in the solid state, excited at a wavelength of 400 nm and showing emission at 469 nm, (b) lifetime analysis plot, (c) EPR spectra of the MOF showing a signal at 2.003 corresponding to radical formation, (d) impedance spectra of MOF under light on-off condition at a frequency range of 1 Hz–1 MHz in 0.2 M Na_2_SO_4_, (e) XPS valence band spectra to find out the HOMO energy level and (f) the band diagram of the MOF in the NHE scale with standard reduction potential values of the CO_2_ reduction product and HOMO–LUMO band position.

The valence band position was obtained from the XPS valence band (Fig. 5e). The valence band position matched the calculations based on the Kubelka–Munk and Mott–Schottky plots. The work function of the material was calculated to be 5.15 eV from the UPS spectra, referenced to the vacuum scale (Fig. S21).^43^ Finally, to construct the overall band diagram of the MOF, the information from the Kubelka–Munk diagram, Mott–Schottky, and XPS spectra was combined (Fig. 5f). The conduction band position of the MOF is more negative than the standard reduction potential for CO_2_ reduction to formate and carbon monoxide, making the reaction feasible. A critical review of the literature revealed that most of the Zr-based MOFs reported for photocatalytic CO_2_ reduction to CO employed costly noble-metal-based salts or photosensitizers to reduce the band gap and shift the conduction band to a range that is feasible to product formation (Table S3). However, this work contributes to new approaches to designing a photo-redox-active MOF that catalyzes the photocatalytic CO_2_ reduction to CO in a simpler, more cost-effective way. Other ways employed are photoactive linker installation, metalation, and salen based complex insertion, *etc.* While comparing with the other reported diimide based scu topology Zr MOF frameworks, the intrinsic narrower channels in this case further enrich CO_2_ concentration at the catalytic centre. In another scu-type Zr MOF NMK-906, Co-Salen functionalization has increased the CO_2_ adsorption drastically, resulting in the effective concentration of CO_2_ molecules near the Co-Salen active sites and leading to a higher production rate of 2.63 mmol g^−1^ h^−1^ of CO.^44^ But such frameworks suffer from reduced mass transfer in the channels. NMK-908 is a csq topology MOF synthesized from the same diimide-based ligand mentioned above, which showed higher CO_2_ adsorption, still a lower production of CO (1.92 mmol g^−1^ h^−1^) along with H_2_ (1.52 mmol g^−1^ h^−1^).^45^ Hence, not only the adsorption but also effective mass transfer and accumulation of small molecules near the catalytic site make the difference in photoreduction. In the current scu framework of study, the micropores are able to channel a good amount of CO_2_ at the catalytic sites without any further modification, probably leading to a good conversion rate.

Furthermore, the reaction mechanism was studied in detail using Raman spectroscopy. The Raman spectrum of the MOF, when collected under 532 nm excitation, did not produce any prominent Raman frequencies. However, when the Raman spectra from the MOF with the CO_2_ solution were collected every 30 seconds of light irradiation and plotted at 5-minute intervals for a total of 50 minutes, it revealed distinct vibrational frequencies corresponding to various reaction intermediates (Fig. 6a). A Raman signal at 1386 cm^−1^ and 1010 cm^−1^ is the characteristic symmetric stretching signal of CO_2_ and HCO_3_^−^, which indicates the proper dissolution of CO_2_ in the reaction medium. The signals at 952 cm^−1^ indicate *CO_2_^−^ species, which is the first active intermediate formed on the catalyst surface during the conversion.^46–48^ Another major intermediate involved in both CO and CH_4_ pathways is *COOH, which showed multiple signals at 1607 cm^−1^ (C=O stretch), 2508 cm^−1^, 801 cm^−1^, and 655 cm^−1^ (O–C=O out-of-plane bending). Along with *COOH, water (H–O–H) bending modes are also present at 1607 cm^−1^.^10,46,48^ Another major peak at 2170 cm^−1^ indicates *CO surface coverage. The signal intensity at 1789 cm^−1^ indicates the formation of a formyl species. C–H stretching at 2802 cm^−1^ and C–H bending or wagging at 1192 cm^−1^ indicate the existence of *OCH_2_ and *OCH_3_ intermediate formation, respectively.^49,50^

**Fig. 6:**
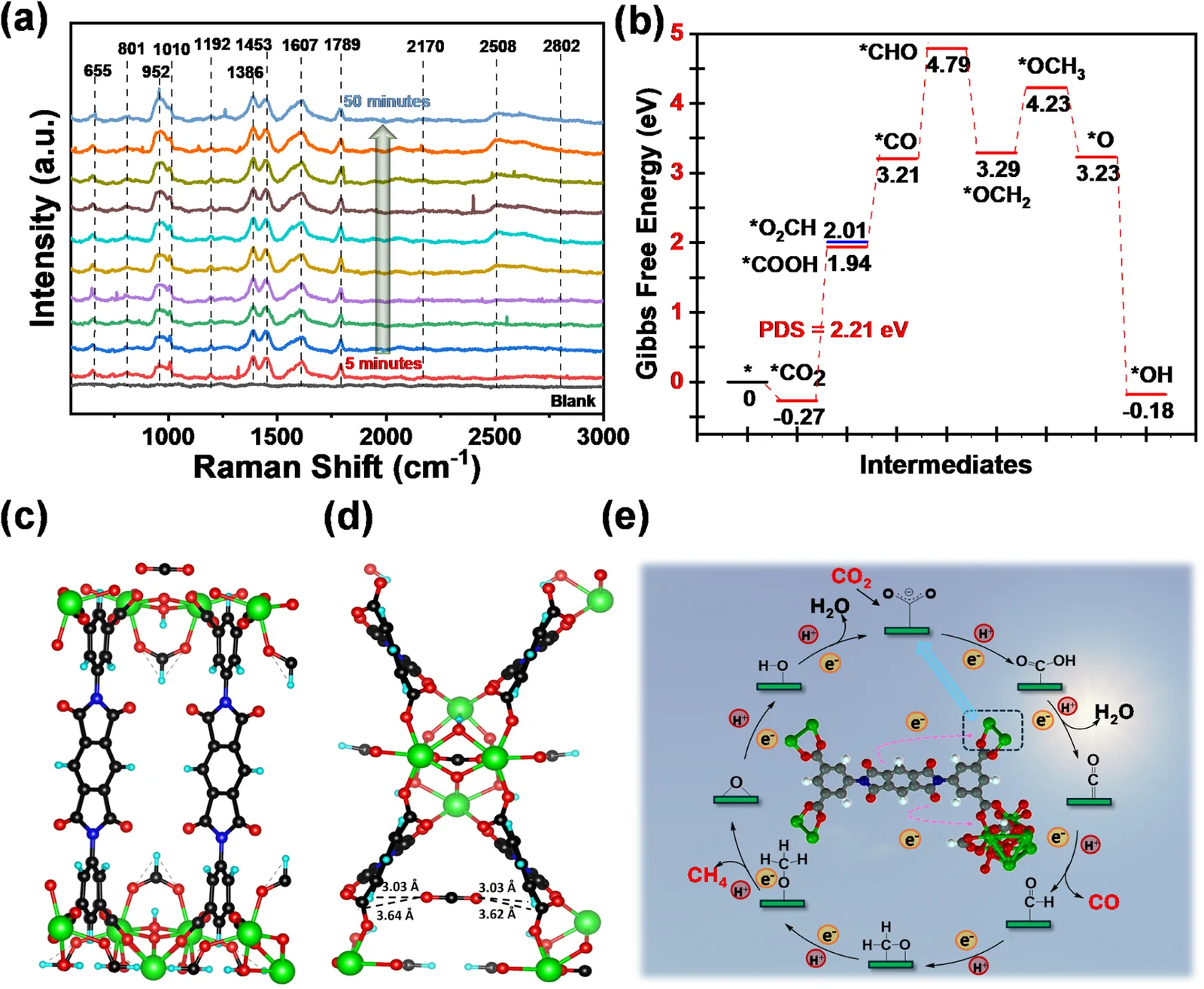
(a) *In situ* Raman scattering showing the stretching frequency of various intermediates, (b) Gibbs free energy profile of the CO_2_ reduction reaction (CO_2_RR) on the Zr-MOF catalyst, comparing the two possible intermediates, (c and d) DFT-optimized ground-state geometry of CO_2_ adsorbed within the pores of the Zr-based MOF: (c) front view, (d) side view and (e) proposed catalytic cycle of CO_2_ photoreduction.

The Gibbs free energy associated with CO_2_ hydrogenation is a critical parameter for catalyst design and optimization (see simulation details in the SI). Effective hydrogenation requires the sequential activation of CO_2_ and H_2_, followed by stabilization of the resulting intermediates. Due to its inherent thermodynamic stability, CO_2_ is challenging to activate and often proceeds through complex pathways. For the Zr-based MOF, the Gibbs free energy for CO_2_ adsorption is calculated to be −0.27 eV. Upon activation, CO_2_ undergoes hydrogenation through two primary intermediates, *O_2_CH and *COOH, with corresponding Gibbs free energies of 2.01 eV and 1.94 eV (Fig. 6b). These values suggest that hydrogenation preferentially proceeds *via* the *COOH pathway to product formation. The energy barrier to form the formyl intermediate from *CO is higher compared to the formation of *CO from *COOH. Hence, we achieved greater selectivity for CO formation. *CO, *CHO, *CH_2_O, *CH_3_O, *O, and *OH, with their optimized ground-state geometries provided in Fig. S22 of the SI. The associated Gibbs free energies are calculated as 3.21 eV, 4.79 eV, 3.29 eV, 4.23 eV, 3.23 eV, and −0.18 eV, respectively. The potential-determining step (PDS) corresponds to the activation of CO_2_ to form the *COOH intermediate, with an energy barrier of 2.21 eV. Similar to the intermediate adsorption in the framework, the CO_2_ adsorption and interactive sites were also investigated. Fig. 6c and d shows the DFT-optimized adsorption geometry of CO_2_ in the Zr-BDI MOF, presented in both top and side views. The CO_2_ molecule is in close proximity to framework hydrogen atoms, with O–H distances of 3.01–3.03 Å and the nearest C–O framework contact of 3.62–3.64 Å. The static binding energy calculated by DFT is approximately −35.25 kJ mol^−1^, consistent with the experimental isosteric heat of adsorption at low loading.

Starting from CO_2_ adsorption to the intermediates observed during photocatalytic reduction, we proposed the most likely pathway (Fig. 6e). During light collection, [ligand]˙^−^ formation diverts the charge accumulation on the catalytic center, activating the CO_2_ molecule.^40^ It takes up a proton–electron pair and is converted into the major *COOH intermediate. Eventually, it loses a water molecule and accepts an electron from the Zr sites, forming a *C=O intermediate. Later, it is released from the catalyst surface as carbon monoxide, while the intermediate continues to accept protons to form the formyl intermediate (*CHO). The intermediate changes its connectivity to the catalyst from carbon to oxygen, passing through *OCH_2_ and *OCH_3_ states, finally releasing CH_4_ from the pathway, thereby leaving a bridged oxygen on the catalyst surface.^47,50^ Thereafter, it takes up two protons and releases a water molecule to regenerate the activated catalyst. While water serves as the proton source in the reaction, the metal centers and the radical-forming ligand contribute to surface charge accumulation.

## Conclusion

This work demonstrates a simple yet effective strategy in which a photo-redox-active linker is used to design a Zr-based, robust MOF for the noble-metal-free conversion of CO_2_ to CO in water/TEOA media. The rational choice of the BDI linker leads to the MOF having a low band gap and favorable conduction band values for CO production. A detailed analysis of the structure–property relationship has been done using both experimental and computational studies. This strategy offers a convenient approach to the future design of MOF-based photocatalysts through the judicious selection of metal and linker building blocks. We believe this work will encourage the design of more MOF catalysts that can convert CO_2_ into other higher liquid and gaseous products.

## Author contributions

ACP conceptualized the work and wrote the manuscript. ACP synthesized and characterized the MOF and did the photocatalytic experiments. AC helped in the photocatalytic experiments. SS helped with the *in situ* Raman studies. AHA simulated the MOF's structure. TSV and RB conducted computational studies to investigate MOF-CO_2_ interactions and reaction intermediates. HA supervised the project, discussed the results, and corrected the manuscript. AN, and SR reviewed and corrected the manuscript.

## Conflicts of interest

There are no conflicts to declare.

## Supplementary Material

RA-OLF-D6RA07959H-s001

## Data Availability

The data supporting this article, titled “Studying the structure–property relationship in a Zr MOF for noble-metal-free photocatalytic CO_2_ reduction: insight on the mechanistic pathway through *Operando* Raman spectroscopy and DFT”, have been included as part of the supplementary information (SI). Any other data will be made available upon request. Supplementary information: additional figures and tables. Ligand characterisation (NMR), cyclic voltammetry, EPR, isosteric enthalpy calculation for CO_2_, high pressure adsorption data for different gases, scanning electron microscopy images, TGA, photocurrent measurements and computational details. See DOI: https://doi.org/10.1039/d6ra07959h.
