# Supplementary material for: Studying the structure–property relationship in Zr-BDI MOF for noble-metal-free selective photocatalytic CO2 conversion to CO
Source: RSC Adv. 2026 Nov 2;16(49):67661–71. doi: 10.1039/d6ra07959h (PMC13725856; doi:10.1039/d6ra07959h)
Supplement: Supplementary file 1 [file RA-016-D6RA07959H-s001.pdf]

Supplementary Information (SI) for RSC Advances.
This journal is © The Royal Society of Chemistry

Studying the structure-property relationship in Zr-BDI MOF for noble-metal-free selective photocatalytic CO_2 conversion to CO

Akhil Chandran P,[a,b] Tushar S. Verma,[c] Shreya Sarkar,[a] Ankur Chanda,[a] Ayalew H. Assen,[d] Amit Nag,[a] Ravichandar Babarao,[c] Sounak Roy *[a,b] and Himanshu Aggarwal* [a,b]

[a]Department of Chemistry, Birla Institute of Technology and Science, Hyderabad Campus, Hyderabad 500078, India. E-mail: himanshu.aggarwal@hyderabad.bits- pilani.ac.in.

[b]Materials Centre for Sustainable Energy & Environment (McSEE), Birla Institute of Technology and Science, Hyderabad Campus, Hyderabad 500078, India.

[c]School of Science, RMIT University, GPO Box 2476, Melbourne, Victoria 3000, Australia.

[d]College of Chemical Sciences and Engineering (CCSE), Applied Chemistry and Engineering Research. Centre of Excellence (ACER CoE), Mohammed VI Polytechnic University (UM6P), Ben Guerir 43150, Morocco.

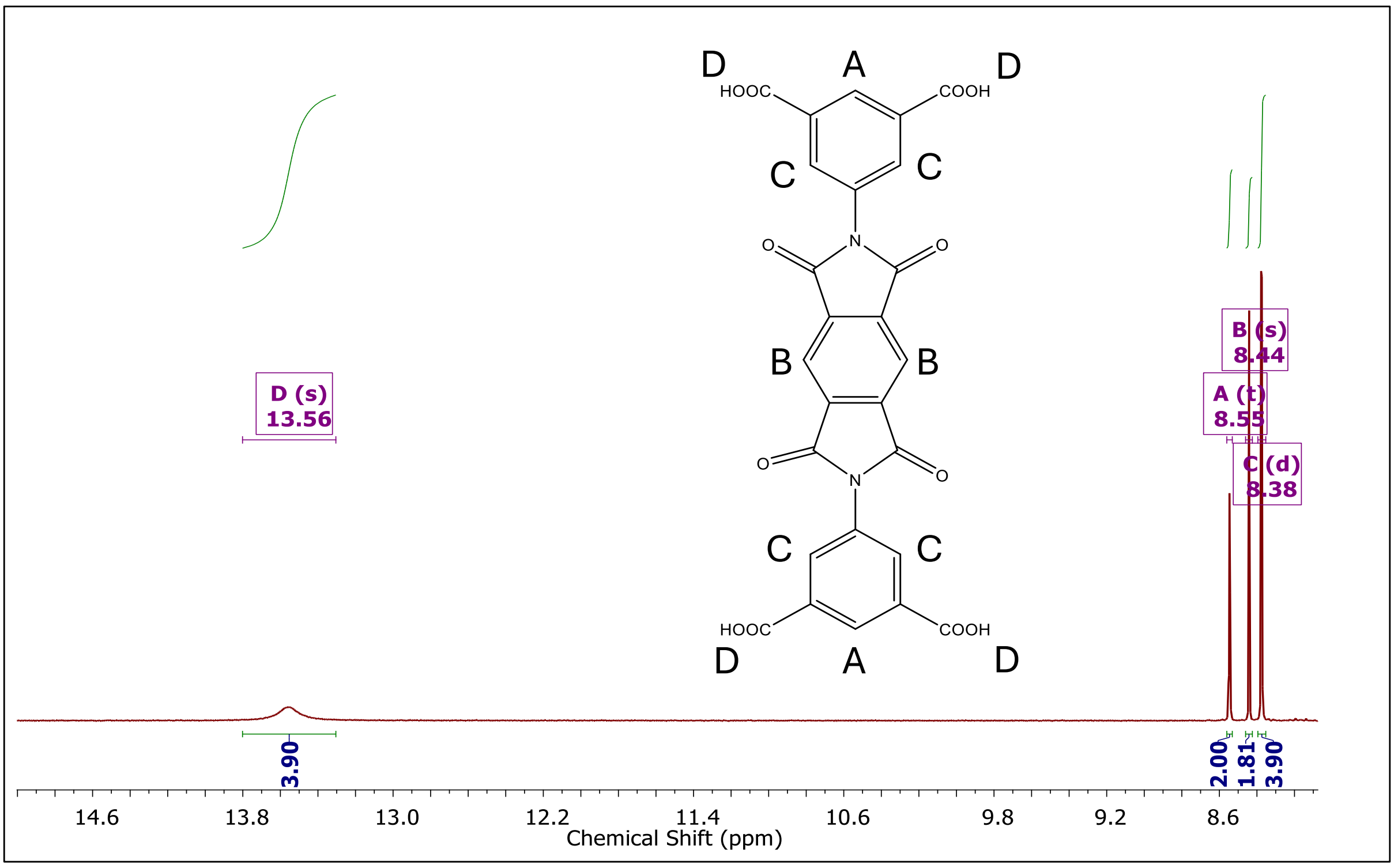

Fig. S1 ^{1}H NMR spectra of the BDI ligand.

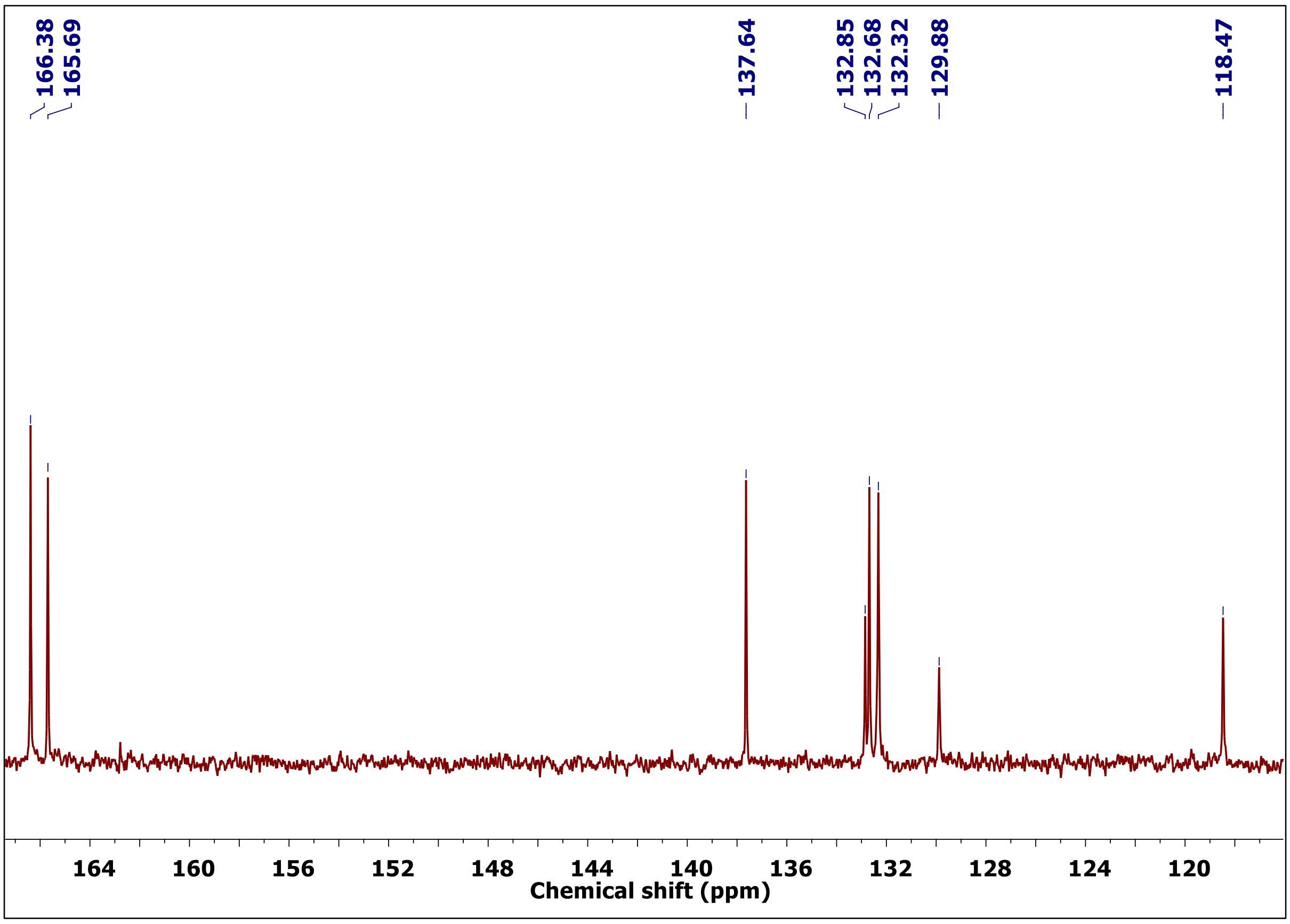

Fig. S2 ^{13}C NMR showing the chemical shift values of the carbon atoms present in the ligand.

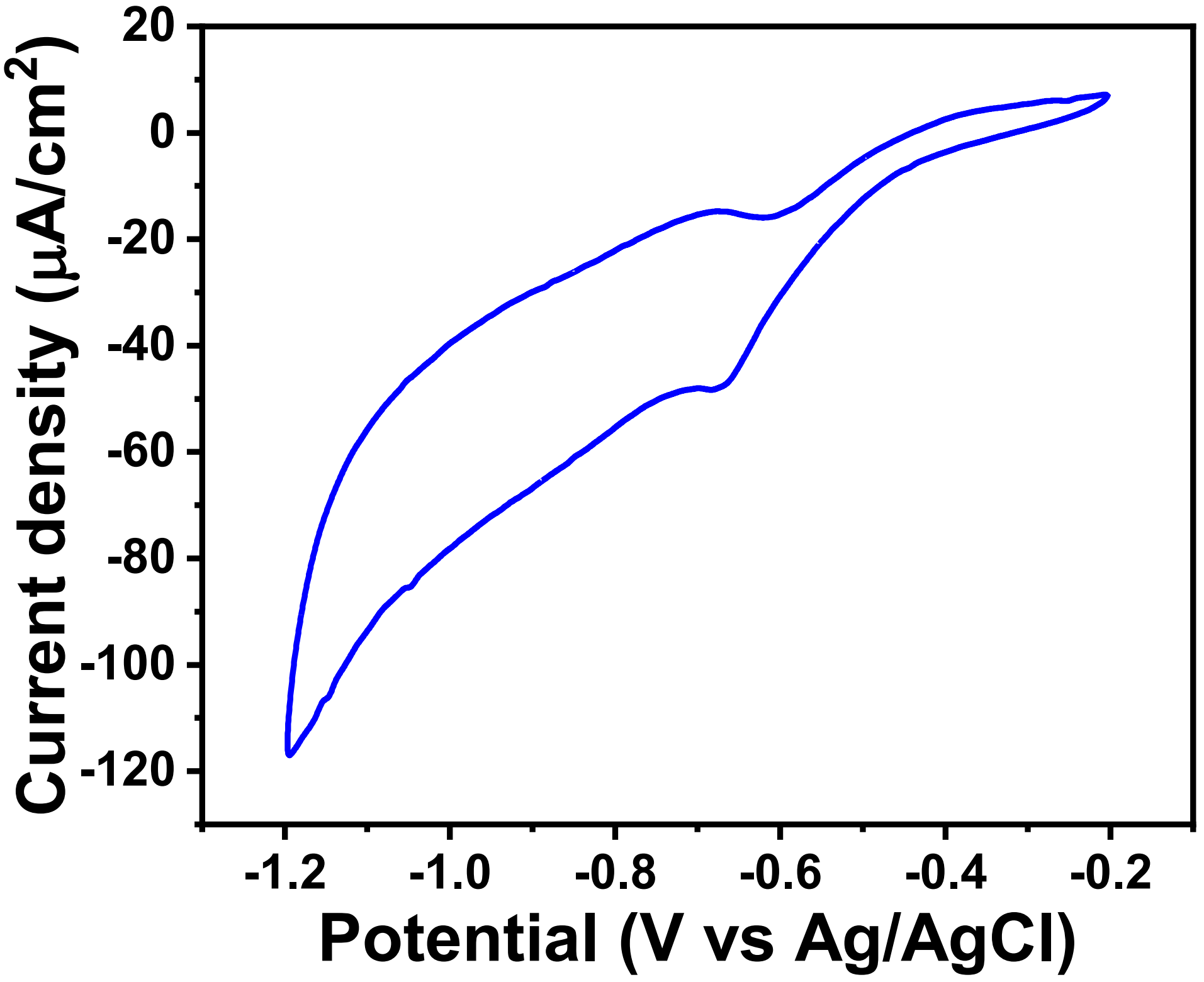

Fig. S3 Cyclic voltammetry of the BDI linker showing redox-active behaviour.

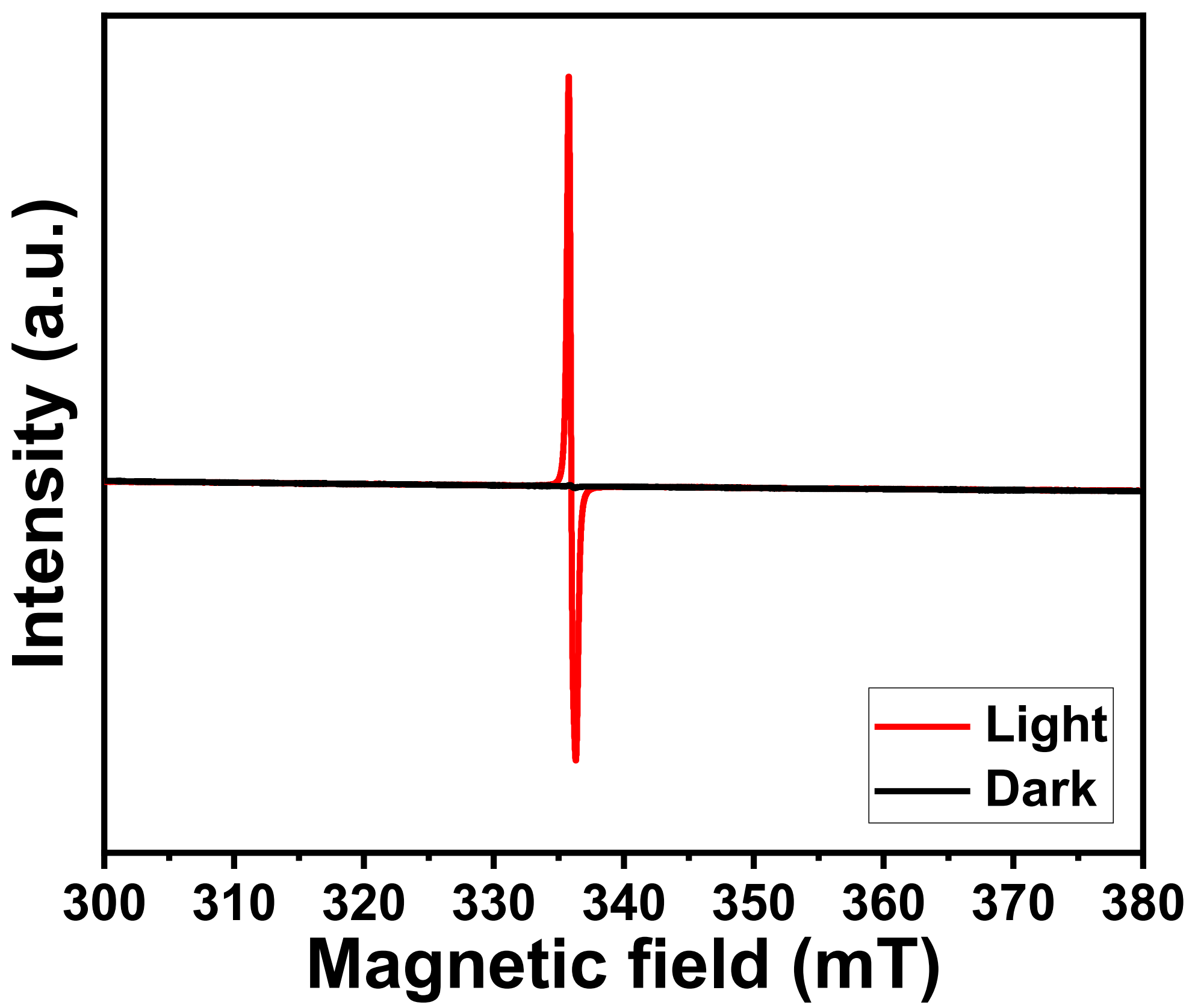

Fig. S4 Ligand shows an EPR signal at g-2.003 in the presence of light proving the redox active behaviour from the cyclic voltammetry.

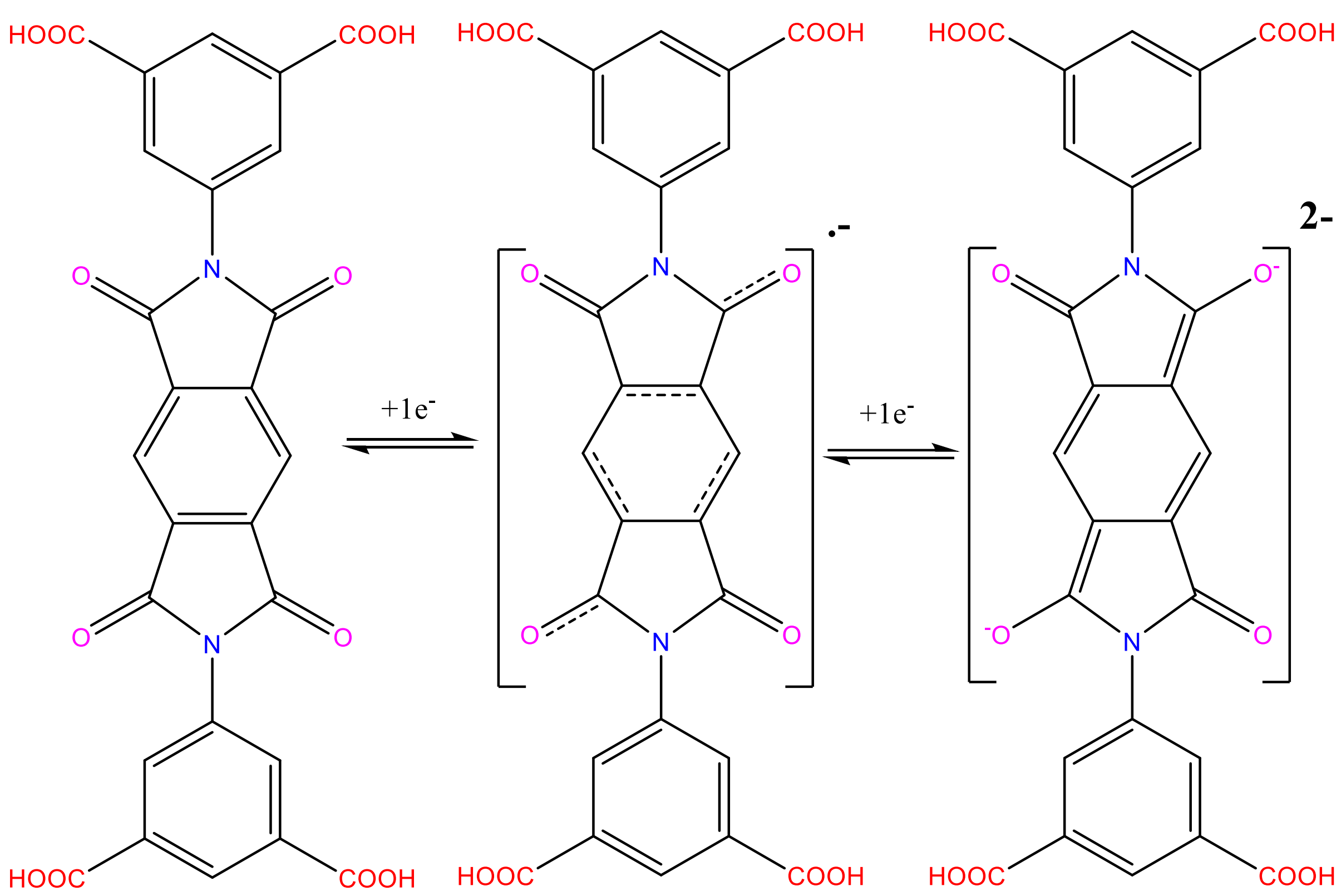

Fig. S5 Redox behaviour of the ligand shows radical formation due to charge transfer.[1]

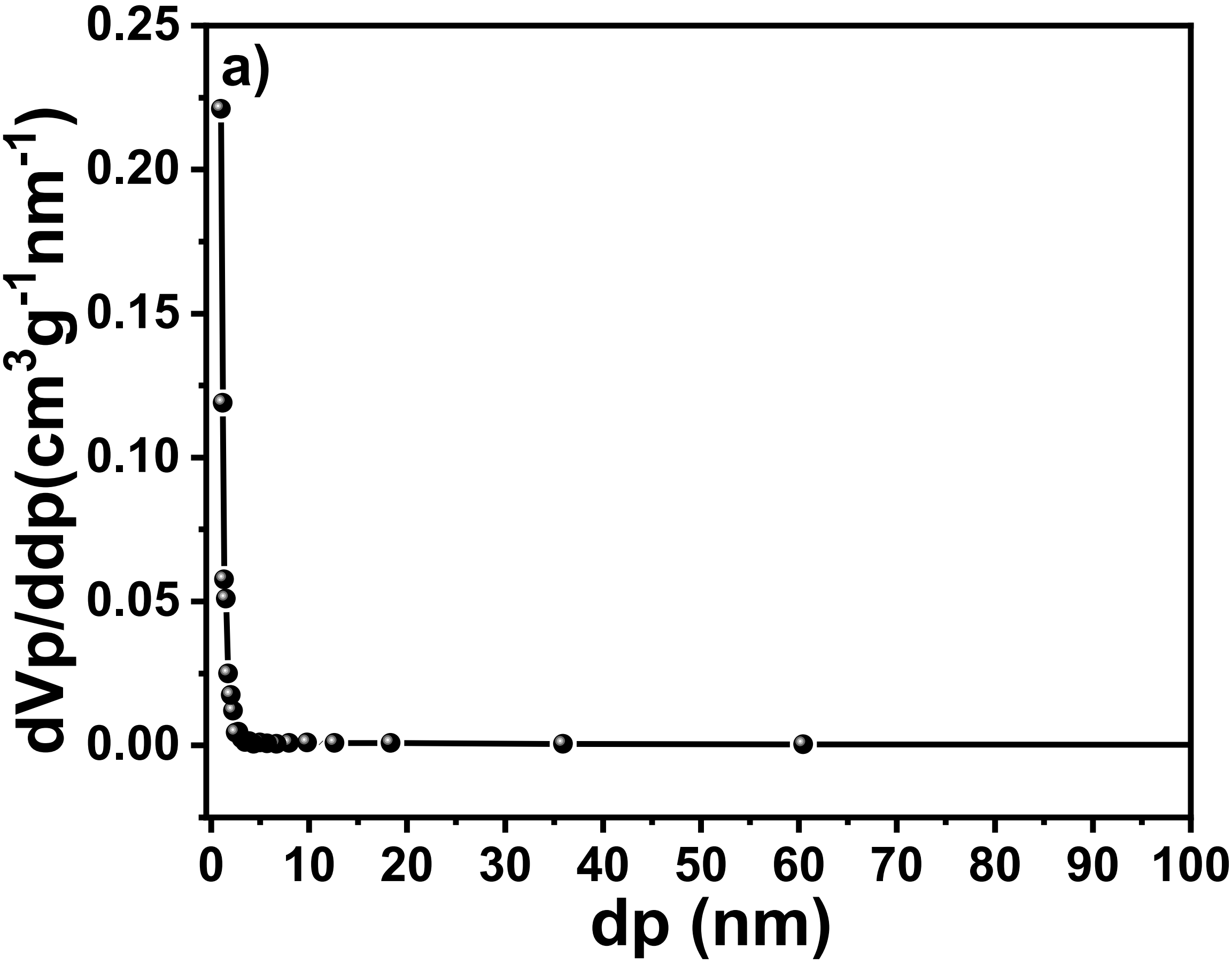

Fig. S6 a) BJH plot confirms the MOF is purely microporous.

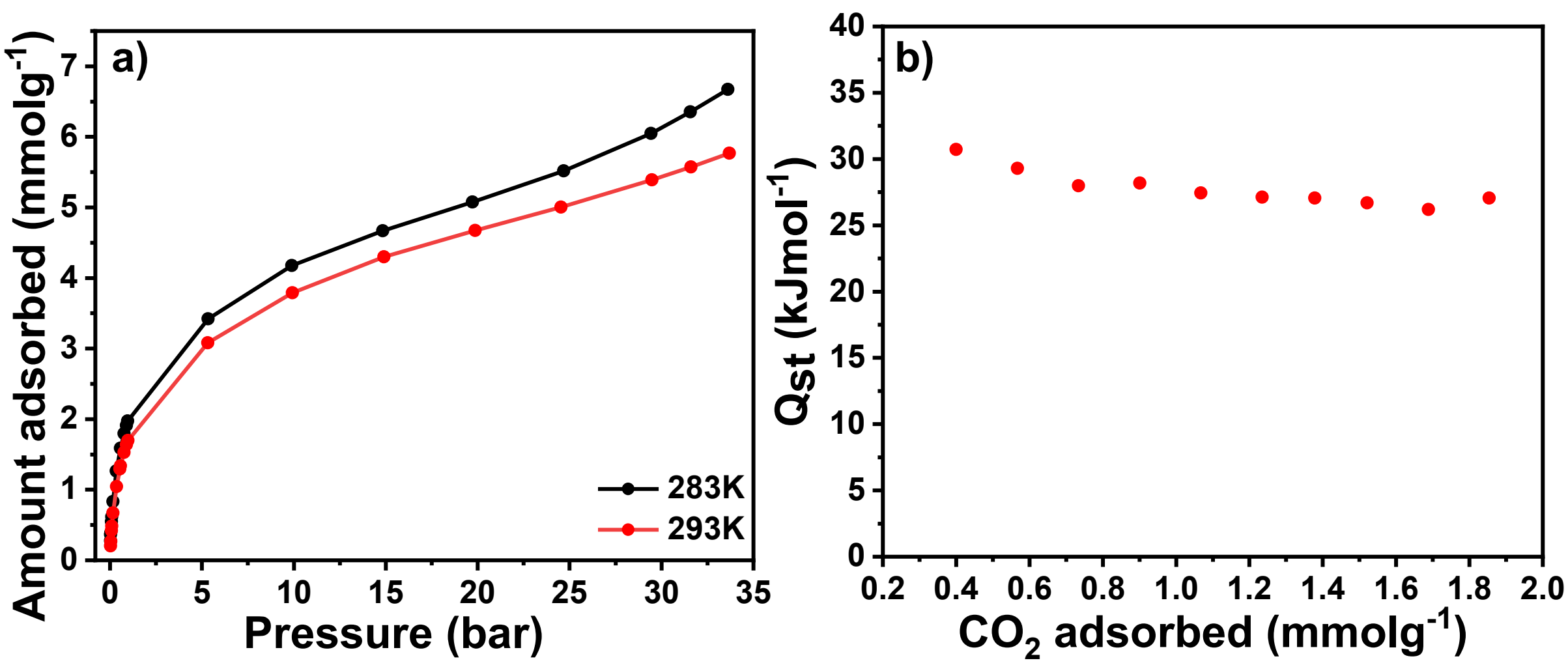

Fig. S7 a) CO_2 uptake of the framework is plotted at two different temperatures for the calculation of the isosteric enthalpy of adsorption, b) Isosteric enthalpy of adsorption plot proving the physisorption interaction of CO_2 with the framework.

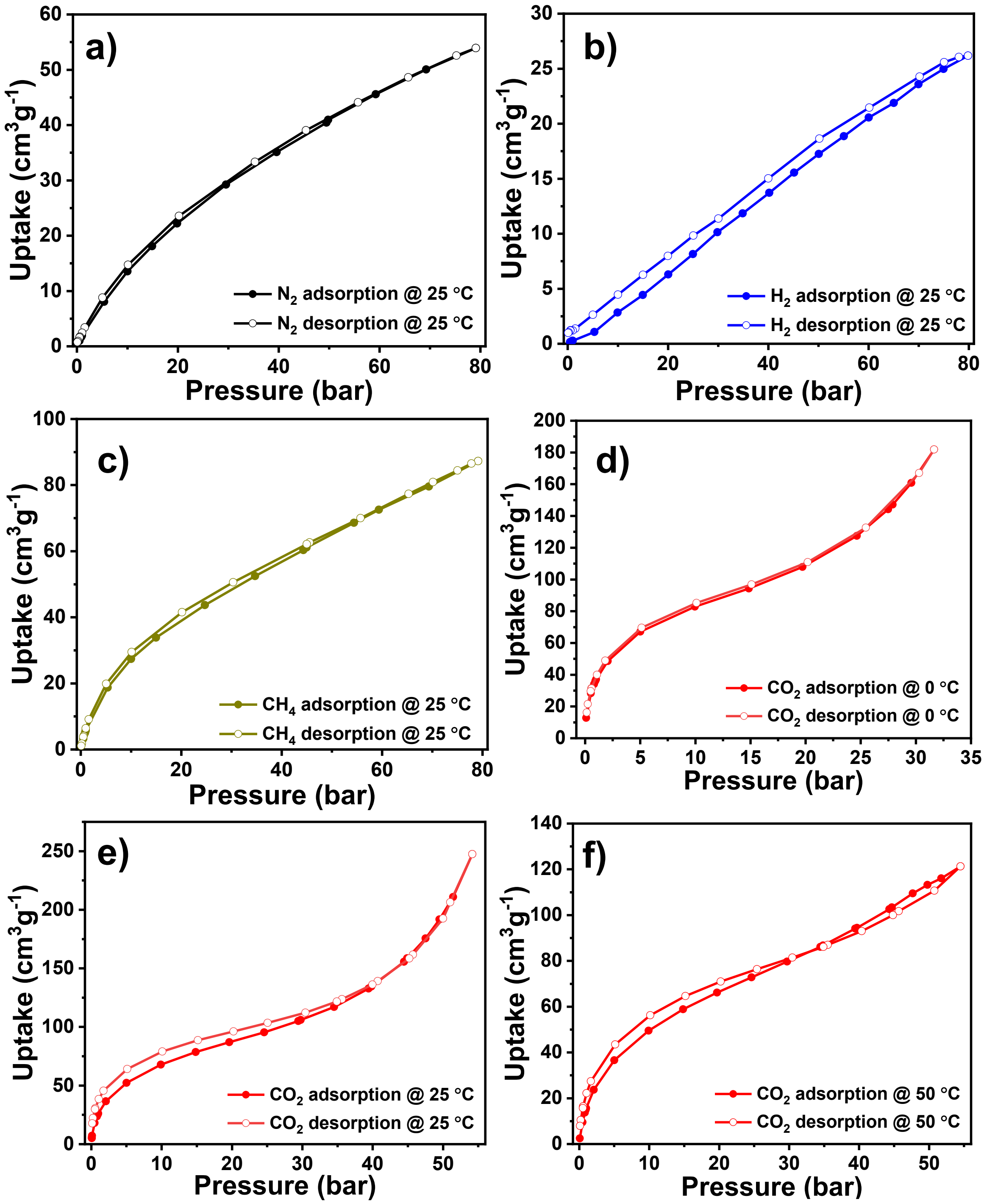

Fig.S8 a-c) Adsorption of different gases at higher pressures and d-f) uptake of CO_2 at different temperatures at higher pressure.

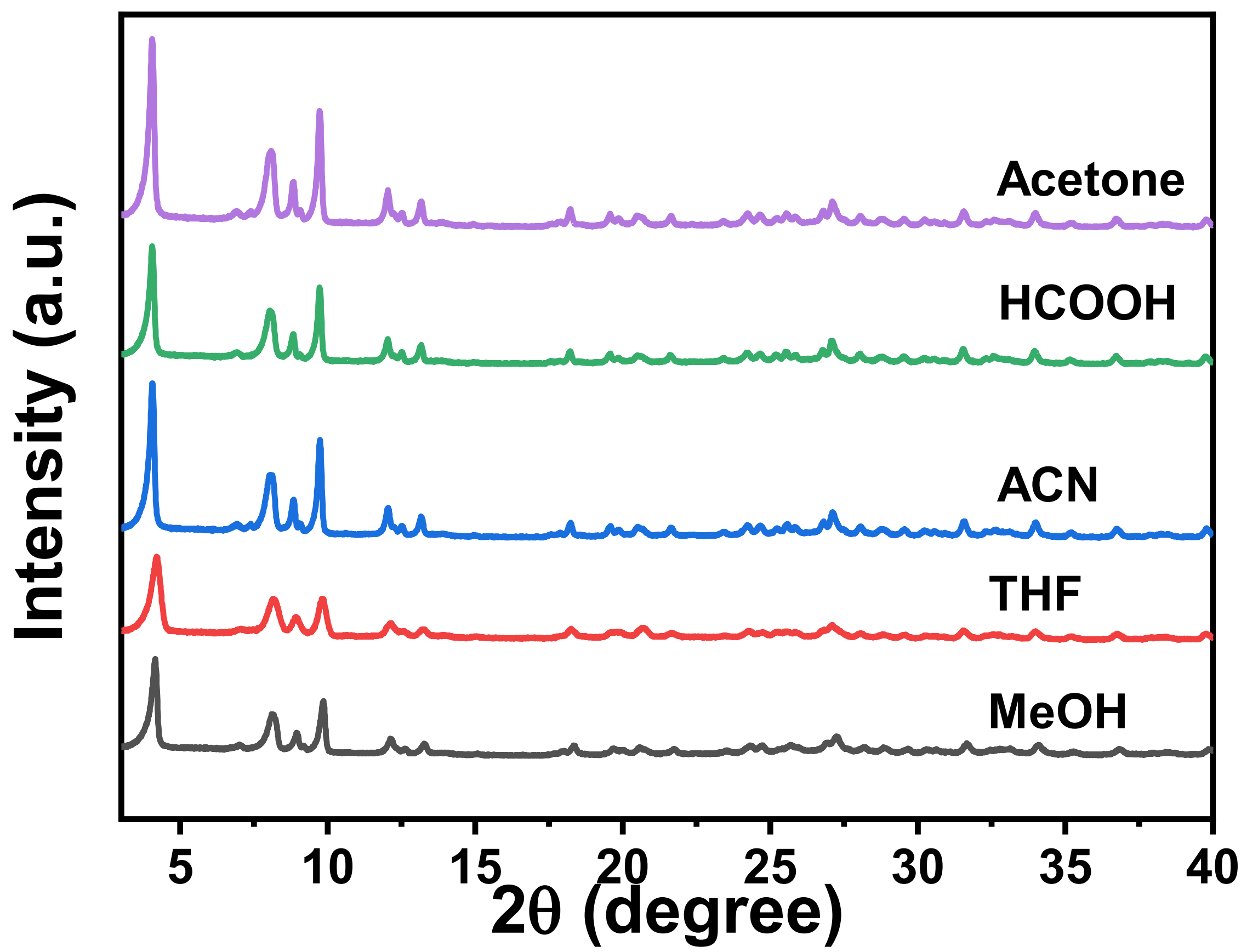

Fig. S9 PXRD pattern of the MOF in different organic media after one week

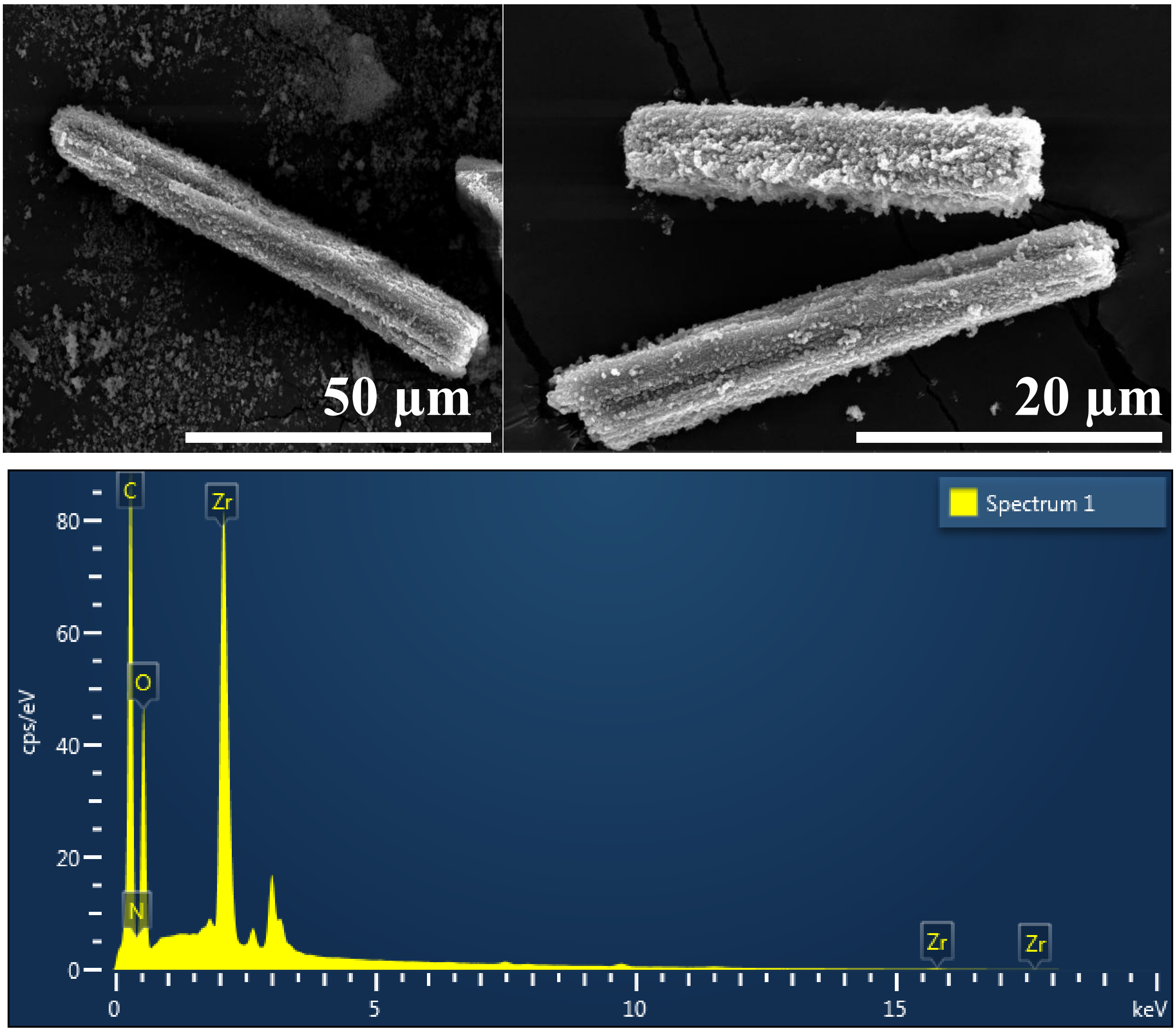

Fig.S10 (top) Rod shaped crystals of the Zr MOF observed in SEM at 50 and 20 µm resolution and (bottom) EDS mapping is done to find out the percentage composition of the elements.

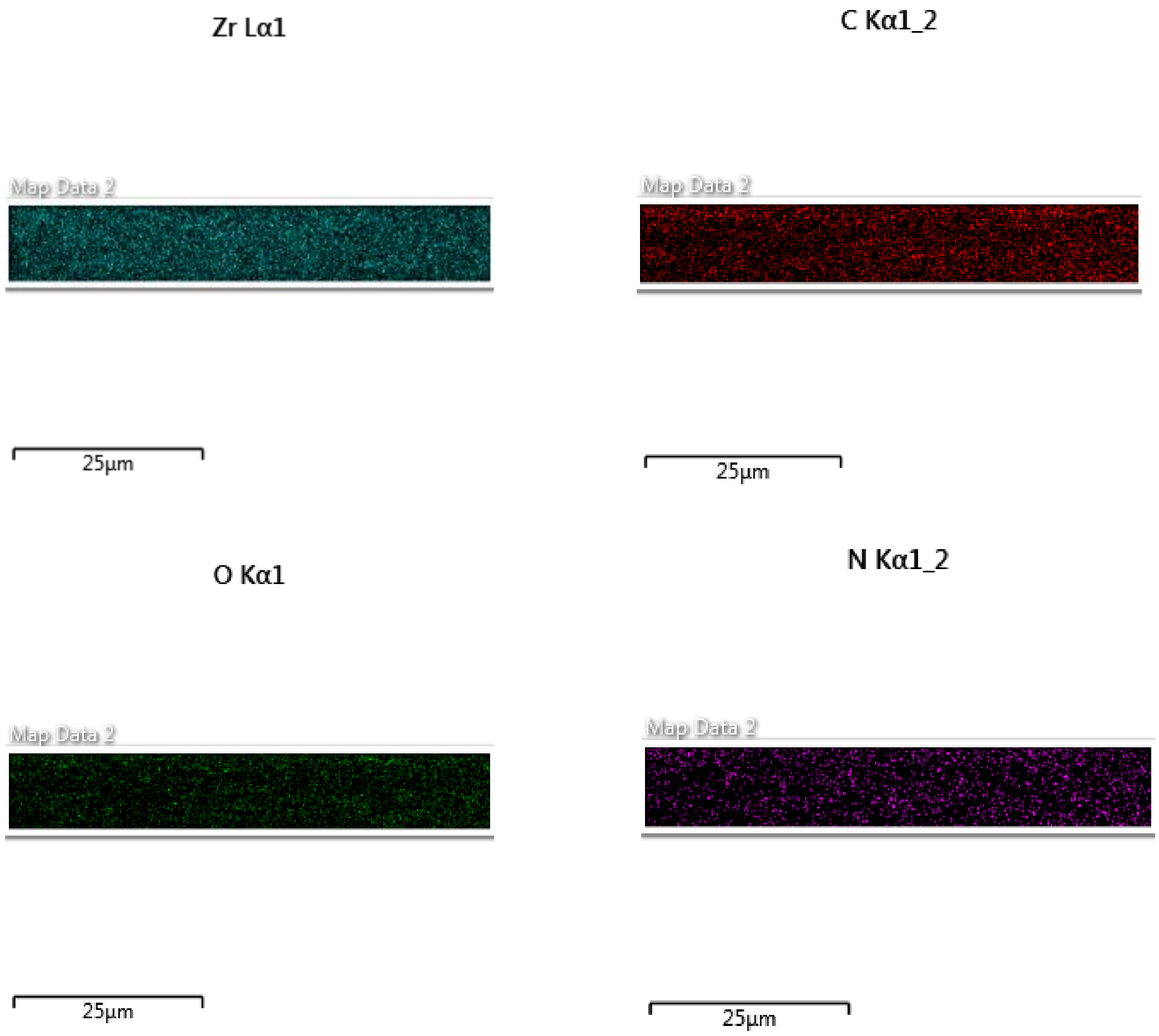

Fig.S11 EDS color mapping tracking different elements in the crystal.

Table. S1 Atomic percentage of the elements obtained from EDS mapping.

Element	Atomic percentage
Zr	4.68
C	68.47
O	25.11
N	1.74

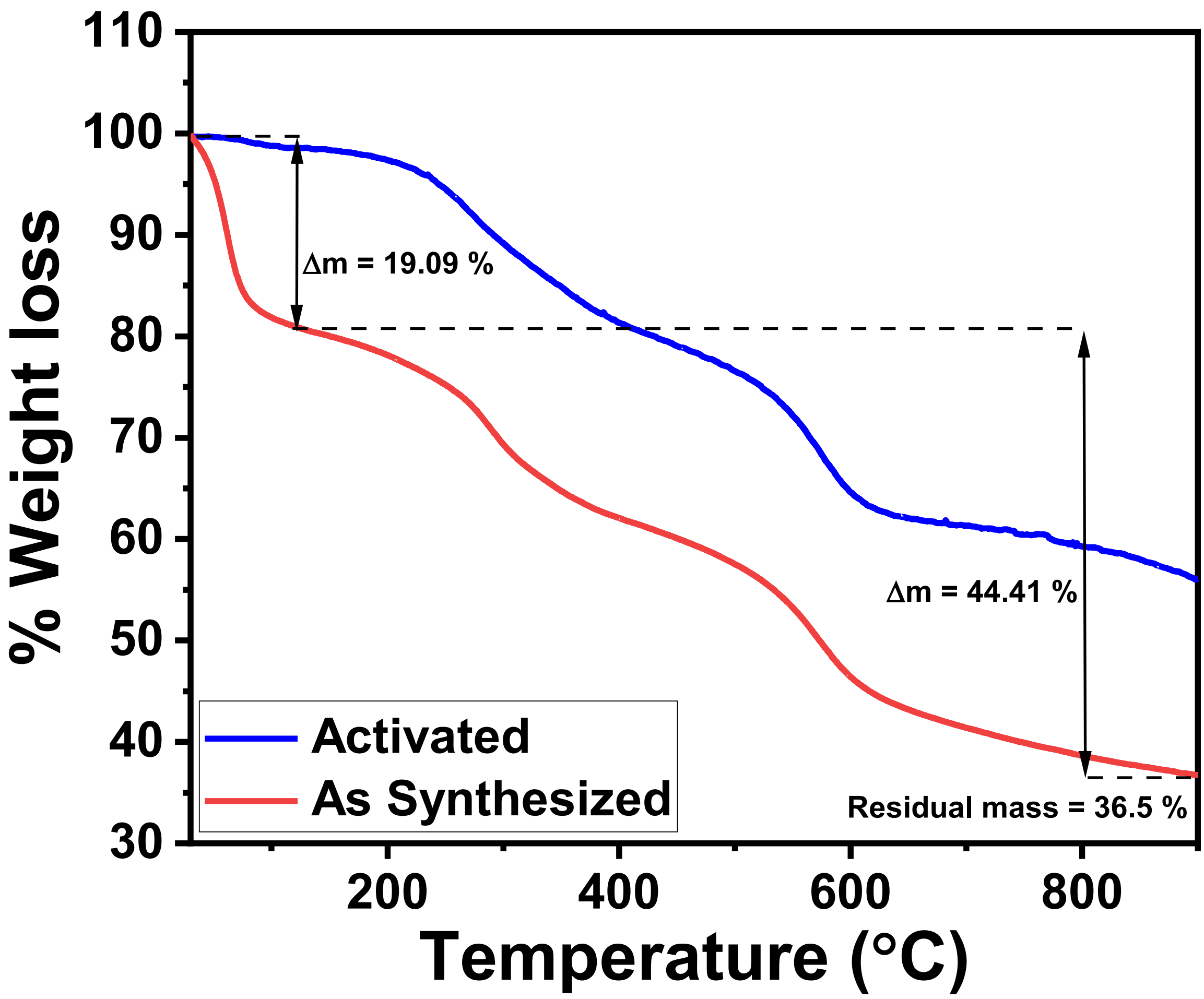

Fig. S12 Comparison between the thermal stability of as-synthesised and activated MOF.

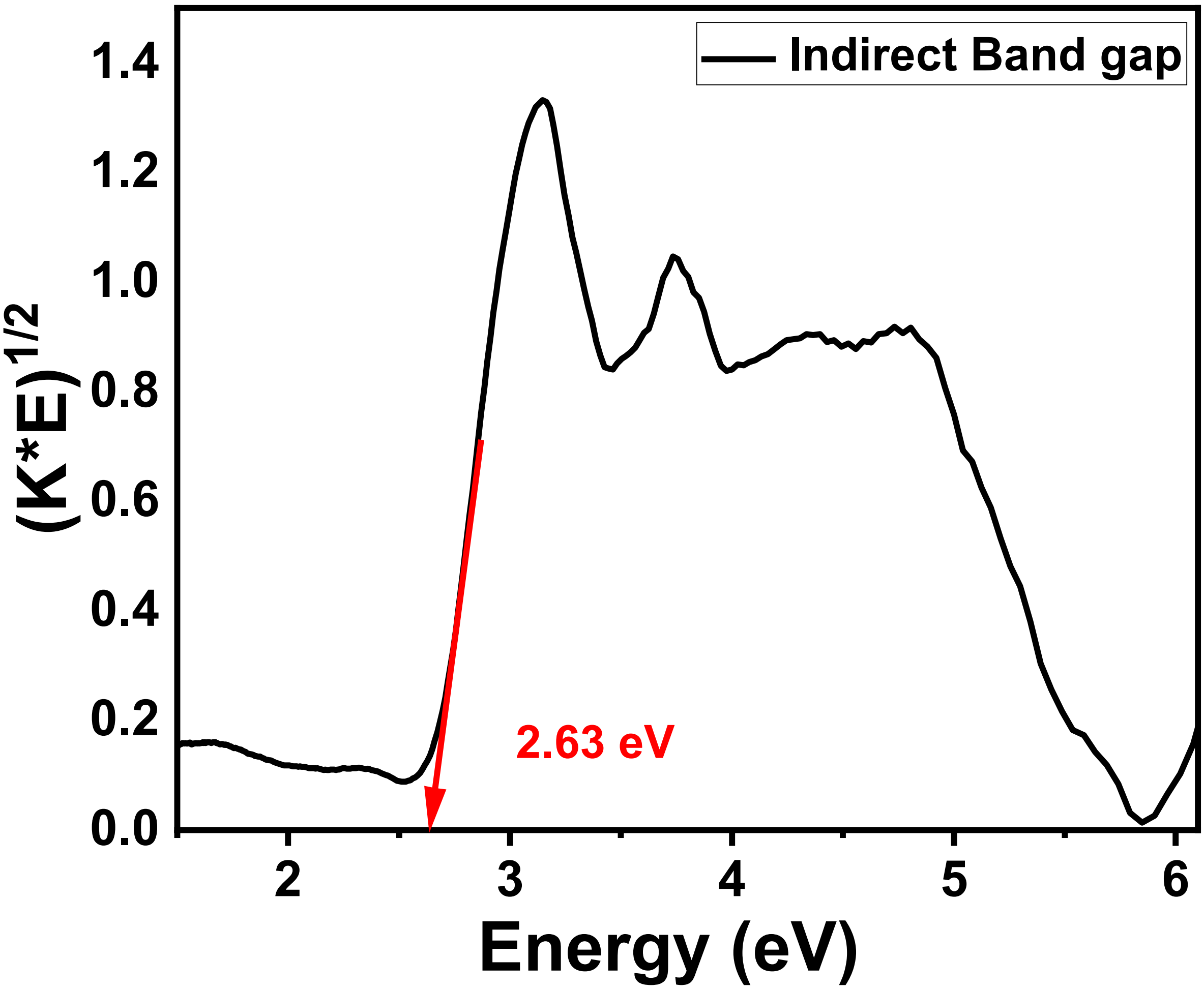

Fig. S13 Indirect band gap of the MOF catalyst from the Kubelka-Munk plot considering the indirect transitions

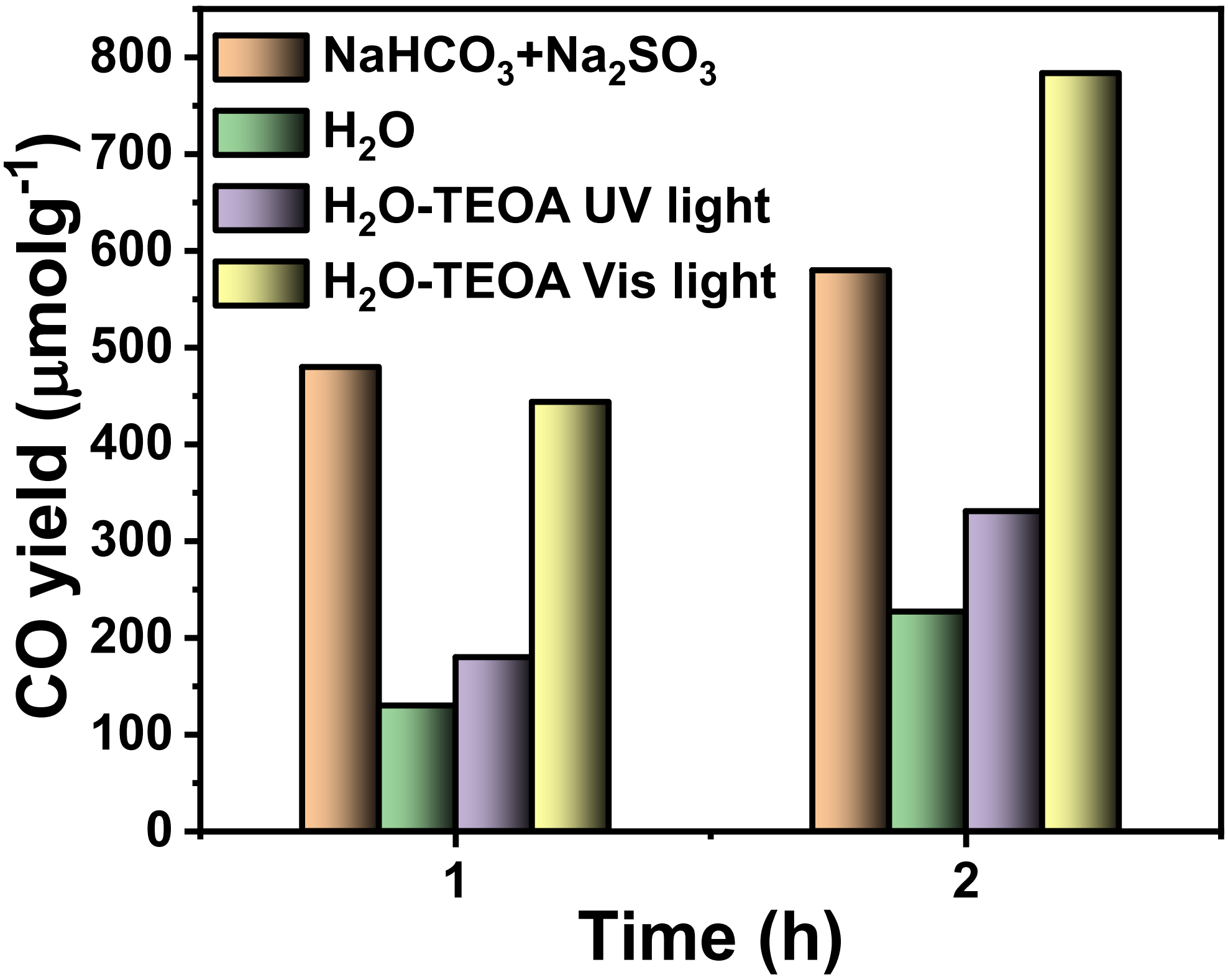

Fig. S14 Photocatalytic reaction carried out at various solvent conditions.

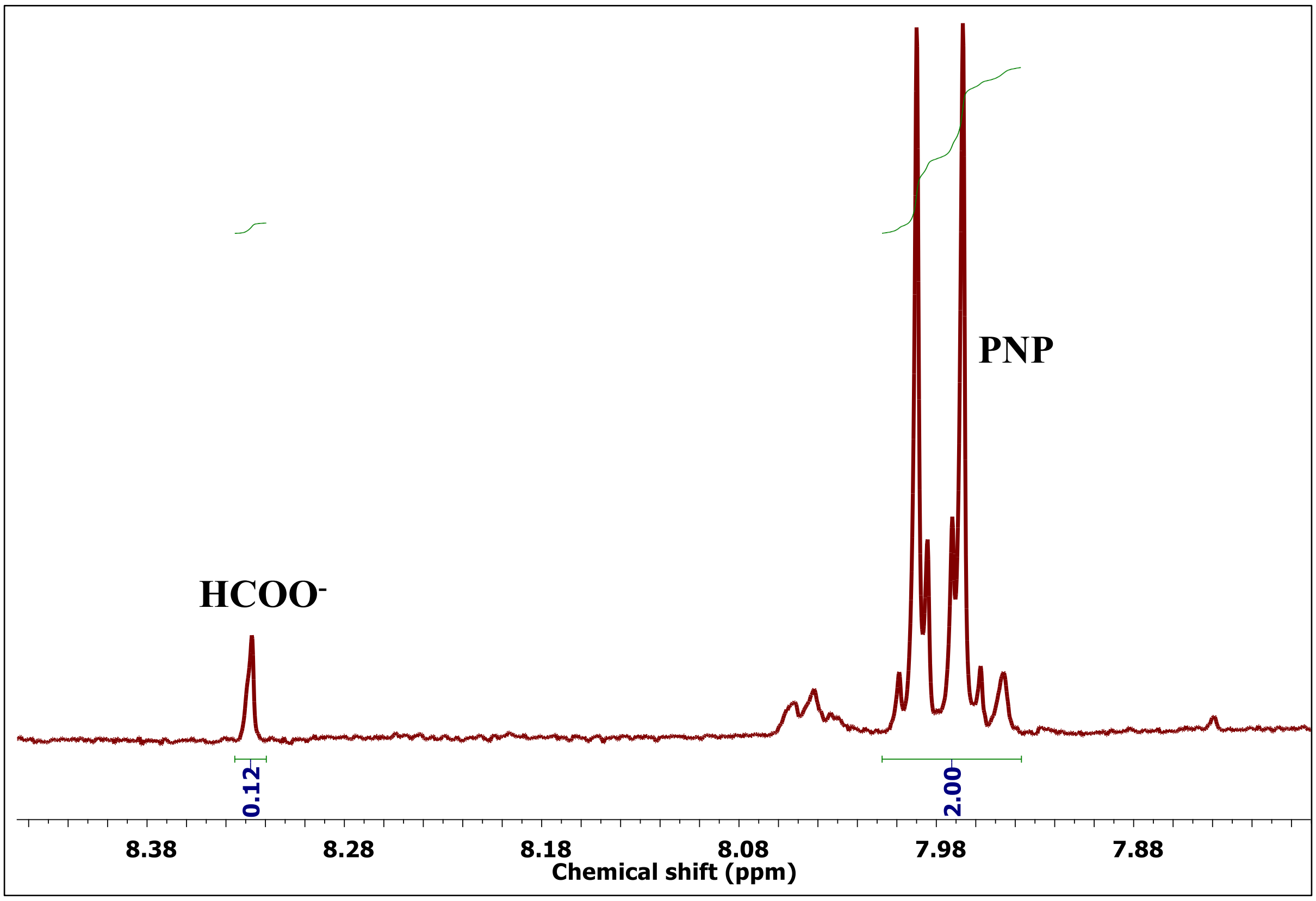

Fig. S15 Product quantification using NMR. The product peak was integrated with respect to the standard and quantified.

NMR quantification:

NMR spectra of the obtained formate after 4h of photoirradiation were integrated.

PNP is the external standard.

From Fig. S19, $[PNP]/[HCOO^-] = [2/2]/[0.12/1] = 8.33$

$[HCOO^-] = [PNP]/8.33$ --(1)

Now, during NMR sample preparation 600 μL aliquot was taken along with 100 μL D_2O and 100 μL external standard (PNP).

Concentration of PNP in the NMR tube is 4.43 mM

From equation 1, we found $[HCOO^-] = 0.531$mM

Hence the amount of $HCOO^- = 0.425$ μmol

Total concentration in 25ml = 13.28 μmol

Production rate from the weight of used catalyst = 265.62 $\mu molg^{-1}$.[2,3]

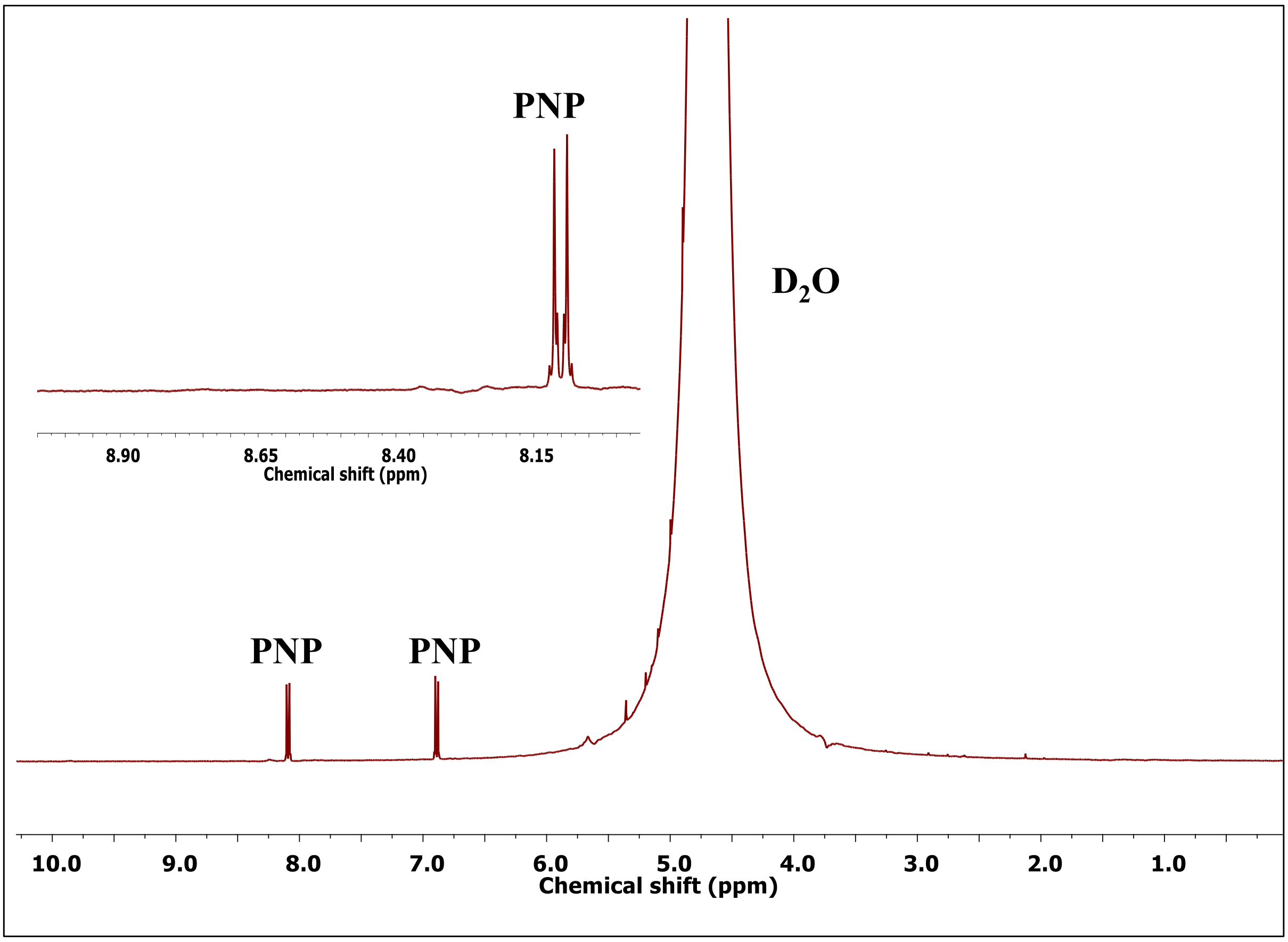

Fig. S16 NMR spectra collected in water after light irradiation for 4h. This proves the formate is not liberated from the coordinated sites of the hexanuclear cluster in the Zr-BDI MOF.

Calculation of selectivity

$$\text{Selectivity of CO} = \frac{[nCO]}{[\text{nCO}]+[\text{nHCOO}-]+[\text{nCH4}]} \times 100\%$$ [4,5]

Table S2. MOF catalysts reported for photocatalytic CO_2 reduction- A comparison

MOF catalyst	Production rate ($\mu molg^{-1}h^{-1}$)	Selectivity	Light source	Reaction medium	Reference

Zr-MBA-Ru/Re-MOF	CO - 440	99	300 W Xe, (λ = 400~800 nm)	H_2O	5
Ti/Zr – MOF - 525	CH_4 - 2.14<br>CO -0.79	NA	150 W Xe lamp (λ>400 nm).	H_2O vapor	6
Co – ZIF - 9/TiO_2	CO - 17.58	62.56	UV - vis (300 W Xe lamp, 200 < λ < 900 nm	H_2O	7
Zr -MBA - Ru/Mn - MOF	CO - 262	99.7	300 W Xe, (λ = 400~800 nm)	H_2O	8
Zr-MBA-TET-Re-MOF	CO - 80.5	99.9	300 W Xe, (λ = 400~800 nm)	H_2O	4
PCN-224 (Cu)/TiO_2	CO - 37.21	NA	300 W Xe lamp (λ > 300 nm)	H_2O	9
NUS-8-$PhSO_3H$	CO - 99.4	100	UV–vis light (320 ~ 780 n m) in Gas-Solid reactor.	NA	10
NNU-28	$HCOO^-$- 52.8	NA	Visible light (420 nm < λ < 800 nm) 300 W Xe lamp	MeCN/TEOA (30:1)	11

PCN - 222	$HCOO^-$ - 60	NA	Visible light (420 nm < λ < 800 nm) 300 W Xe lamp	MeCN/TEOA (10:1)	[12]
UiO-66 (Zr/Ti)-NH_2	$HCOO^-$ - 148.2	NA	Visible light (420 nm < λ < 800 nm) 300 W Xe lamp	MeCN/TEOA (4:1), 0.1M BNAH	[13]
NH_2-MIL-125(Ti)	$HCOO^-$ - 16.28	NA	Visible light (420 nm < λ < 800 nm) 500 W Xe lamp	MeCN/TEOA (5:1)	[14]
1-Ti_{30}	$HCOO^-$ - 71	NA	UV-Vis 125 W medium pressure Hg vapor lamp (200nm < λ < 400nm)	0.1M $NaHCO_3$ / 0.2M Na_2SO_3	[15]
**Zr BDI**	**CO – 270.8 $HCOO^-$-66.40**	**CO – 72.12 $HCOO^-$-17.6**	**Visible light 250W High pressure Hg vapor lamp (400 nm < λ < 800 nm)**	**Water/TEOA**	**This work**

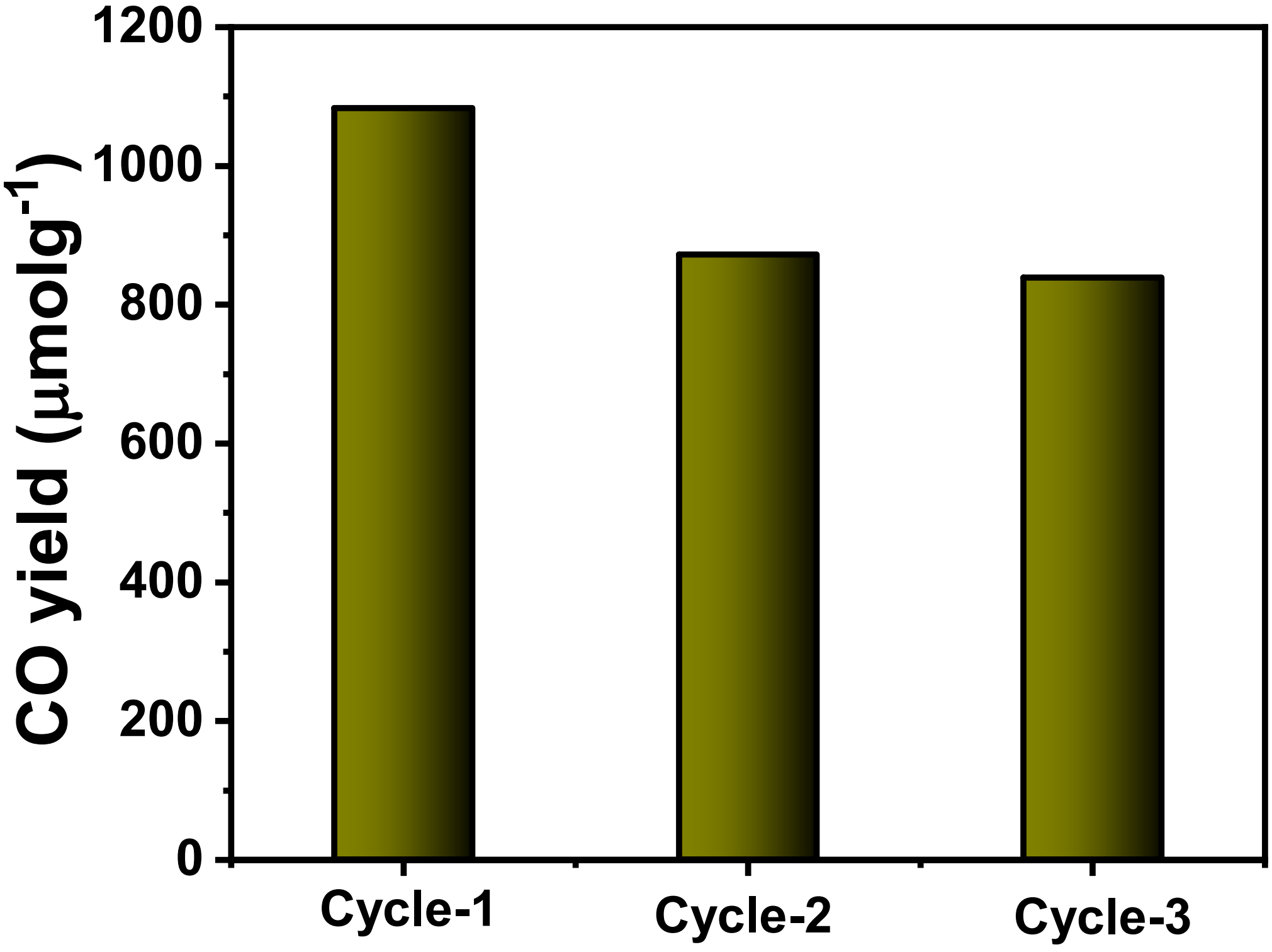

Fig.S17 Cyclic stability shown by the catalyst. After each cycle, the catalyst was separated from the medium and reactivated and used for further cycle.

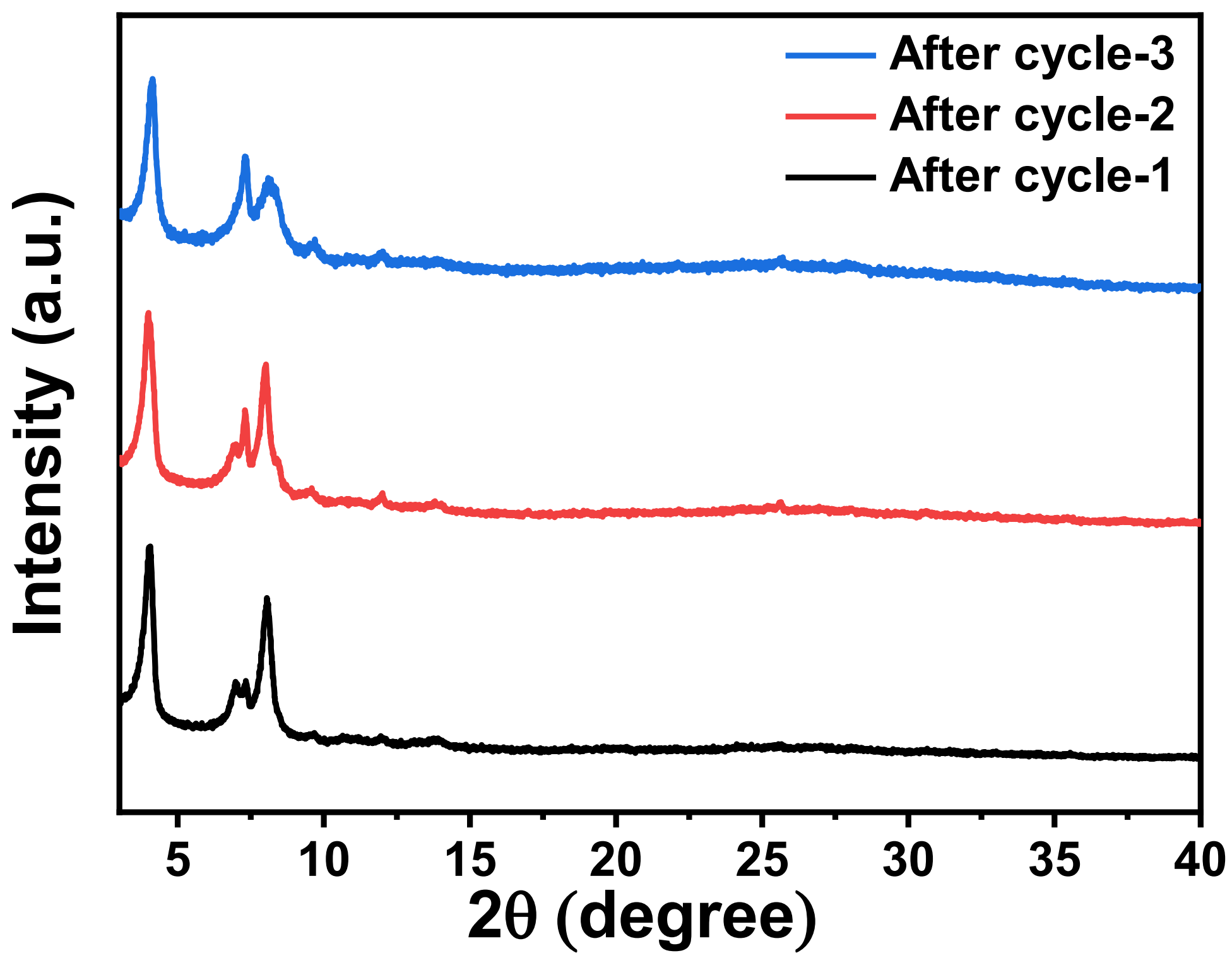

Fig. S18 PXRD of the used sample in the reaction.

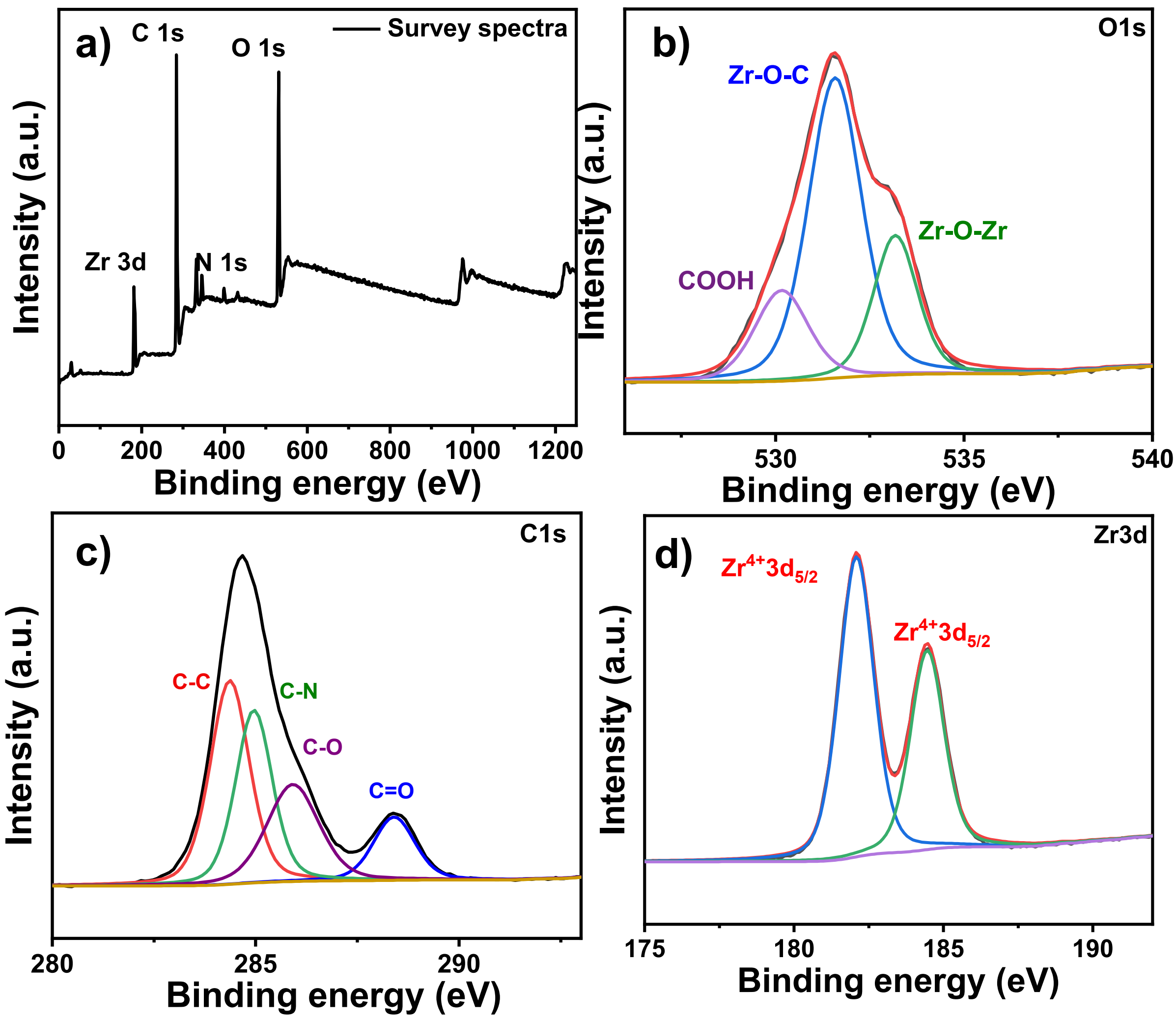

Fig. S19 XPS spectra collected after the third cycle. a) Survey spectra b) O1s spectra c) C1s spectra d) Zr 3d spectra.

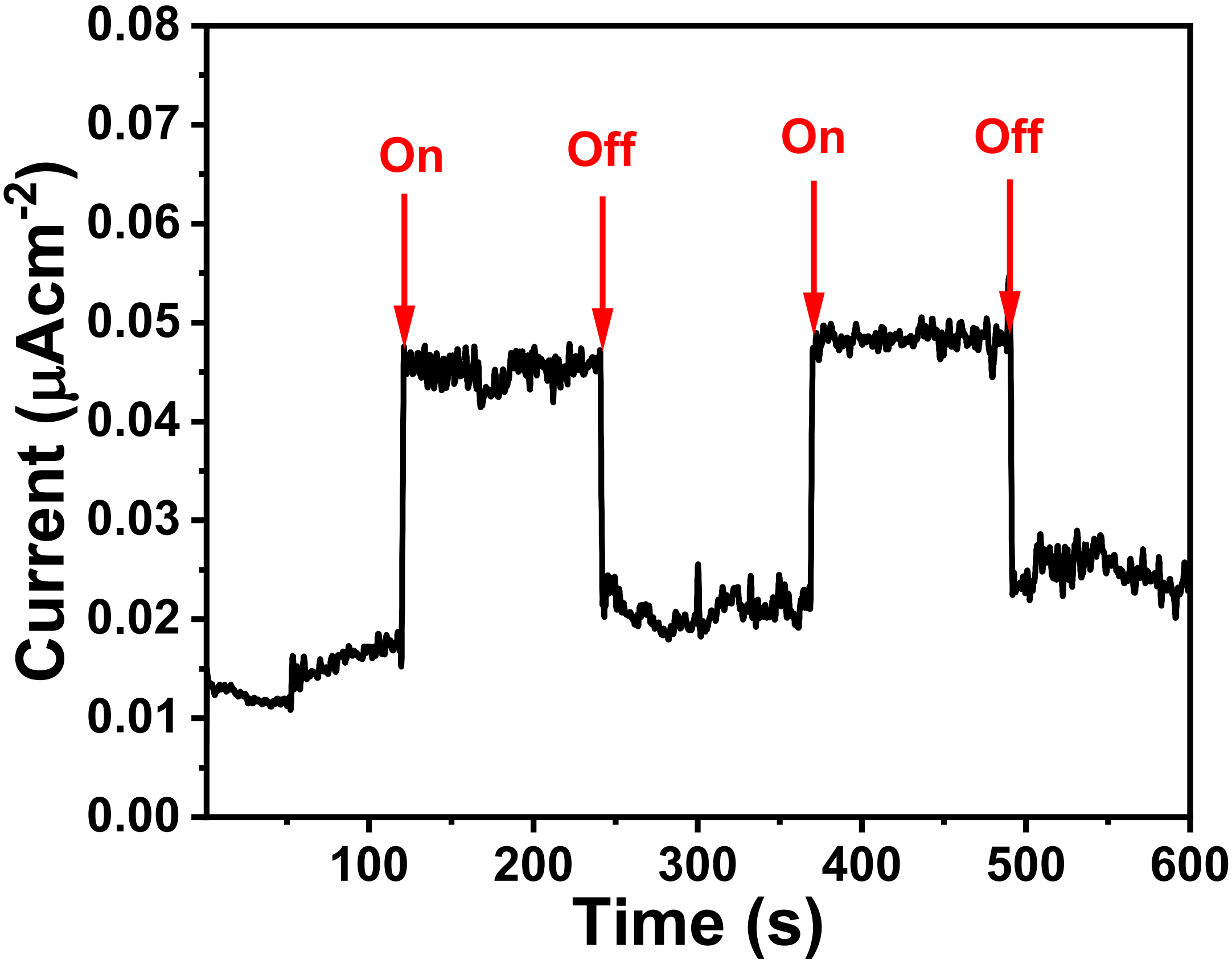

Fig. S20 Transient photocurrent experiment showing the response of the MOF towards light under on-off conditions.

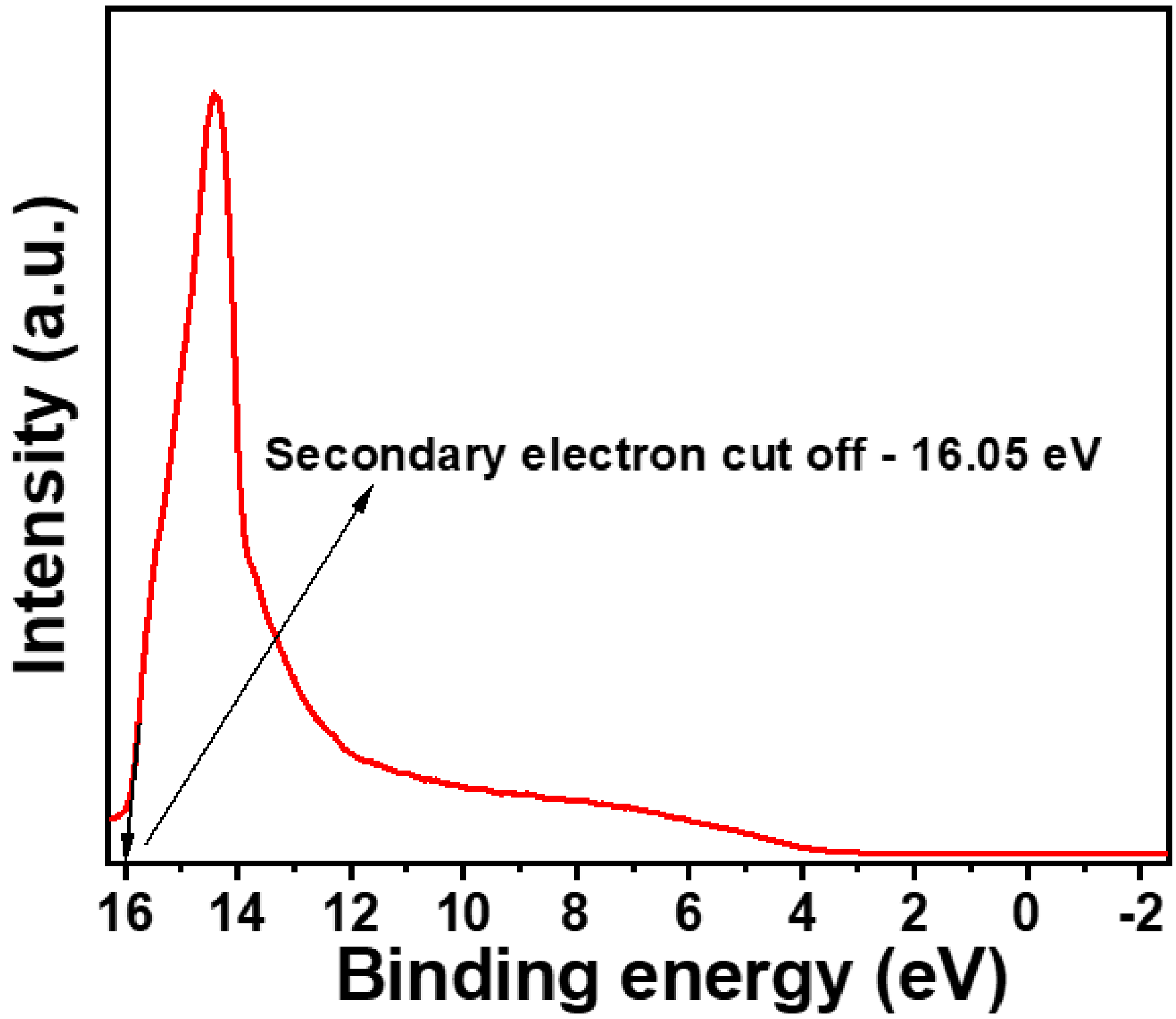

Fig. S21 UPS spectra of the MOF.

Table S3. Band properties of MOF catalysts reported for photocatalytic CO_2 reduction- A comparison

MOF	Band gap (eV)	Modified band gap (eV)	Conduction band<br>Vs. NHE	Maximum Yield	Reference
MOF-808(Zr-MBA-Ru/Re-MOF)	4.06	2 eV	-0.62 V	CO-1580 µmol g^{-1}	5
MOF-808 (Zr-MBA-Ru/Mn-MOF)	4.06	2.67 eV	-0.627 V	CO-1027 µmol g^{-1}	8

MOF-808-PBA-MV	4.06	1.93 eV	-0.67 V	CH_4-7300 µmol g^{-1}	4
Zr-MBA-TET-Re-MOF	4.06	2.09 eV	-0.583 V	CO-362.7 µmol g^{-1}	16
Ru@MOF-808	4.06	-	-	CO-382 µmol$h^{-1}g^{-1}$	17
Zr-DMBD−Co MOF	-	-	-	CO-3.33 µmol	18
1) Zr-PTTB PC,<br>2) Zr/Co-PTTB PC<br>3) Zr/Ni-PTTB PC	1) 2.60	2) 1.85, and 3) 1.86 eV	1) −0.74, 2) −0.70, and 3) −0.63 V	CO-293.2 µmol g^{-1} h^{-1}	19
1) NH_2-UiO-66-Ac<br>2) NH_2-UiO-66-$Fc_{(0.5)}$<br>3) NH_2-UiO-66-$Fc_{(1.0)}$	3.85	1) 2.87<br>2) 2.85<br>3) 2.84<br>4) 2.78<br>5) 2.77	1) −0.66<br>2) −0.94<br>3) −0.98<br>4) −1.03<br>5) −1.06	CO-90.65 µmol $\cdot g^{-1}\cdot h^{-1}$	20

4) NH_2-UiO-66-$Fc_{(1.5)}$<br>5) NH_2-UiO-66-$Fc_{(2.0)}$<br>6) NH_2-UiO-66-$Fc_{(2.5)}$		6) 2.74	6) −1.10		
**Zr BDI**	**2.84 eV**	**-**	**-0.64**	**CO- 270.8 µmol $g^{-1}h^{-1}$**	**This work**

Discussion on simulation

Location of CO_2

The initial positions of CO_2 molecule within the Zr-MOF structure were determined using the classical simulated annealing technique implemented in the Sorption module of *Materials Studio*.[21] During the simulations, the framework atoms were kept fixed, while a single gas molecule was introduced into a simulation box composed of multiple unit cells, ensuring that each dimension was at least twice the cutoff radius. Gas–framework interactions were described by a combination of pairwise site–site Lennard-Jones (LJ) and Coulombic potentials, with LJ parameters for both framework atoms and gas molecules taken from the Universal Force Field (UFF).[22]

In the simulated annealing procedure, the temperature was decreased stepwise, enabling the gas molecule to relax into favourable configurations through configurationally biased NVT Monte Carlo moves, including translation, rotation, and repositioning, each assigned preset probabilities. This heating–cooling cycle was repeated multiple times to locate local minima. A total of forty annealing cycles were performed, with the maximum and final temperatures set at 105 K and 100 K, respectively. The lowest-energy configuration obtained from simulated annealing was selected as the initial structure for subsequent DFT interaction energy calculations.

Interaction Energies

Static interaction energies (ΔE) at 0 K in vacuum were calculated using the following expression:

$$\Delta E = E_{MOF+gas} - E_{MOF} - E_{gas}$$

where E_X refers, respectively, to the total energies of the MOF + gas complex, the MOF alone, and the gas, respectively.

DFT calculations

Kohn–Sham generalized DFT was employed for all geometry optimizations, with Grimme's semi-empirical DFT-D3 corrections applied to account for van der Waals interactions.[23-25] Exchange–correlation energies were described using the Perdew–Burke–Ernzerhof (PBE) functional within the generalized gradient approximation (GGA), as implemented in VASP (version 5.4.4).[26] The PBE functional is widely adopted in solid-state simulations due to its balance between accuracy and computational efficiency. For Zr-based MOF, a 2 × 1 × 1 Γ-point grid was used for geometry optimizations. Electron–ion interactions were described using the projector augmented wave (PAW) method.[27] An energy cutoff of 900 eV was employed during cell shape and size optimizations to minimize volume-dependent basis set effects, followed by a 520 eV cutoff for subsequent plane-wave basis calculations. Γ-point sampling was applied for Brillouin zone integration. Convergence criteria were set to 10^{-7} eV for electronic energy and 0.005 eV $Å^{-1}$ for atomic forces.

The change in gibbs free energy (ΔG) between two adjacent intemediates can be calculated by –

$$\Delta G = G_{final} - G_{initial} \quad \ldots(1)$$

For periodic investigations, $G = E + ZPE - TS + G_{T.C.}$ …(2)

Where, G = Gibbs free energy

E= Electronic Energy of stable intemediate

ZPE = Zero Point Energy of intermediate

TS = Vibrational Entropy of intermediate

$G_{T.C.}$ = Thermal Correction in Gibbs Free Energy of intermediate

All terms are calculated from frequency calculations of adsorbed gas species. ZPE of each intermediate is give by - ZPE = ½ (Σ hν) calculated from non-negative vibrational frequencies, Vibrational frequency (TS) is solved by the formula –

$$S = -R\ \{\Sigma \ln (1 - e^{-h\nu\ kT})\} + R\ \Sigma\ h\nu\ kT\ \{e^{-h\nu\ kT} / (1 - e^{-h\nu\ kT})\}$$

where, R=8.314J·mol^{-1}K^{-1}, k =1.38*10-23J·K^{-1}, h=6.63*10-34J·s and T=298.15 K, ν is the vibrational frequency.

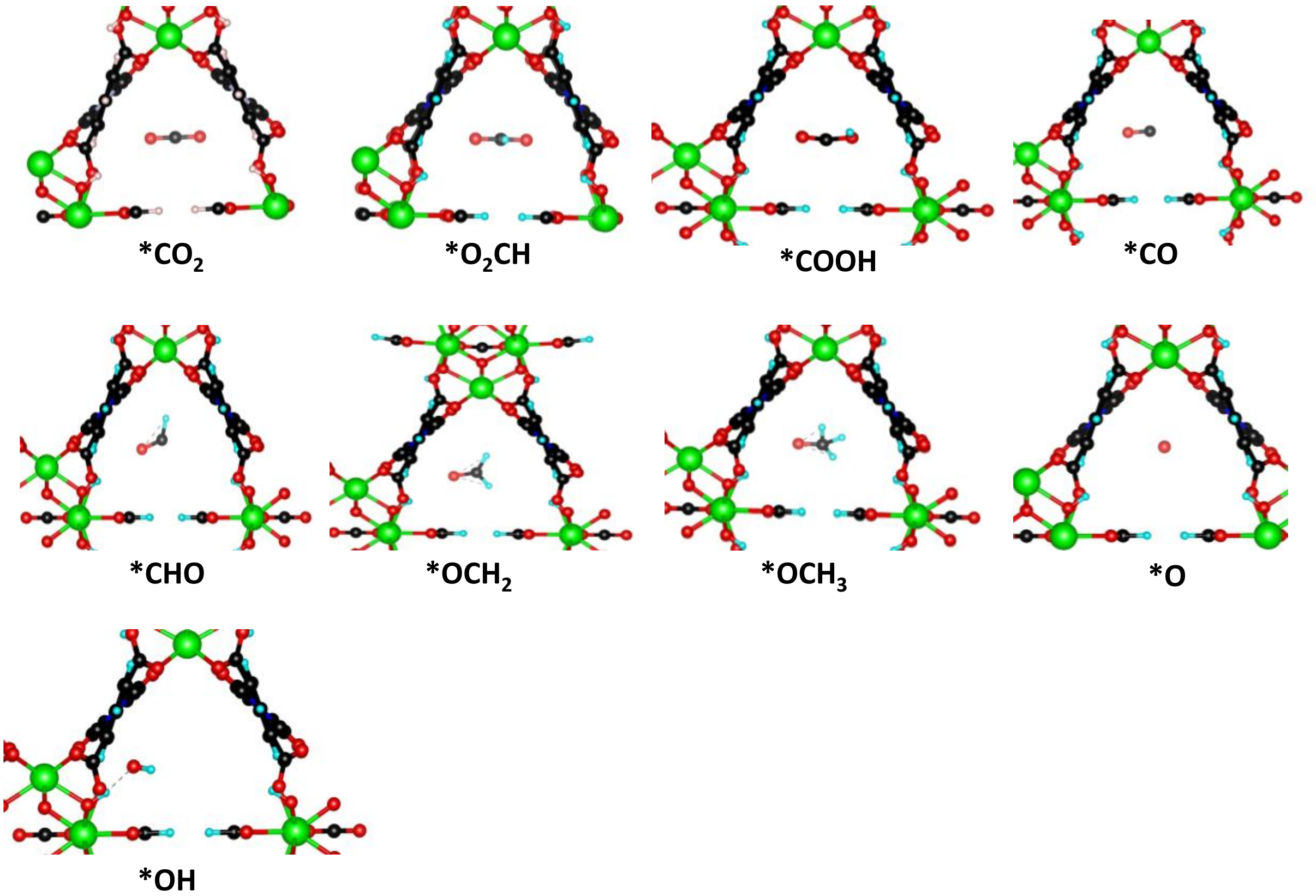

Figure S22. DFT optimized ground-state geometries of key intermediates. Cluster cleaved from the periodic cell for clarity.

References

(1) A. Mallick, H. Liang, O. Shekhah, J. Jia, G. Mouchaham, A. Shkurenko, Y. Belmabkhout, H. N. Alshareef, M. Eddaoudi, *Chem. Commun.,* Made-to-Order Porous Electrodes for Supercapacitors: MOFs Embedded with Redox-Active Centers as a Case Study, 2020, **56**, 1883–1886.

(2) S. Karmakar, S. Barman, F. A. Rahimi, S. Biswas, S. Nath, T. K. Maji, *Energy Environ. Sci.,* Developing Post-Modified Ce-MOF as a Photocatalyst : A Detail Mechanistic Insight Product Formation., 2023, **16**, 2187–2198.

(3) A. Singh, S. Barman, F. A. Rahimi, A. Dey, R. Jena, R. Kumar, N. Mathew, D. Bhattacharyya, T. K. Maji, *Energy Environ. Sci.* Atomically Dispersed Co^{2+} in a Redox-Active COF for Electrochemical CO_2 Reduction to Ethanol: Unravelling Mechanistic Insight through Operando Studies., 2024, **17**, 2315–2325.

(4) S. R. V. Parambil, F. A. Rahimi, R. Ghosh, S. Nath, T. K. Maji,. *Inorg. Chem.* Pore-Confined π-Chromophoric Tetracene as a Visible Light Harvester toward MOF-Based Photocatalytic CO_2 Reduction in Water, 2023, **62**, 19312–19322.

(5) S. Karmakar, S. Barman, F. A. Rahimi, T. K. Maji, *Energy Environ. Sci.* Covalent Grafting of Molecular Photosensitizer and Catalyst on MOF-808: Effect of Pore Confinement toward Visible Light-Driven CO_2 reduction in Water., 2021, **14**, 2429–2440.

(6) W. Y. Gao, H. T. Ngo, Z. Niu, W. Zhang, Y. Pan, Z. Yang, V. R. Bhethanabotla, B. Joseph, B.Aguila, S. Ma, *ChemSusChem.* A Mixed-Metal Porphyrinic Framework Promoting Gas-Phase CO_2 Photoreduction without Organic Sacrificial Agents., 2020, **13**, 6273–6277.

(7) S. Yan, S. Ouyang, H. Xu, M. Zhao, X. Zhang and J. Ye, Co-ZIF-9/TiO_2 Nanostructure for Superior CO_2 Photoreduction Activity. *J. Mater. Chem. A.* 2016, **4**, 15126–15133.

(8) S. R. V. Parambil, S. Karmakar, F. A. Rahimi, T. K. Maji, Confining Molecular Photosensitizer and Catalyst in MOF toward Artificial Photosynthesis: Validating Electron Transfer by In Situ DRIFT Study. *ACS Appl. Mater. Interfaces.* 2023, **15**, 27821–27831.

(9) L. Wang, P. Jin, J. Huang, H. She, Q. Wang, Integration of Copper(II)-Porphyrin Zirconium Metal-Organic Framework and Titanium Dioxide to Construct Z-Scheme System for Highly Improved Photocatalytic CO_2 Reduction. *ACS Sustain. Chem. Eng.* 2019, **7**, 15660–15670.

(10) X. Huang, Z. Wang, Y. Zhong, Y. Jiang, S. Chen, J. Chen, S. Deng and J. Wang, Versatile Strategy for Broadening Light Absorption to Ultraviolet–Visible Region on Zr-Based MOF Photocatalysts for Efficient CO_2 Reduction. *Chem. Eng. J.* 2025, **507**, 160812.

(11) D. Chen, H. Xing, C. Wang, Z. Su, Highly Efficient Visible-Light-Driven CO_2 Reduction to Formate by a New Anthracene-Based Zirconium MOF via Dual Catalytic Routes. *J. Mater. Chem. A.* 2016, **4**, 2657–2662.

(12) H. Xu, J. Hu, D. Wang, Z. Li, Q. Zhang, Y. Luo, S. Yu and H. Jiang, Visible-Light Photoreduction of CO_2 in a Metal − Organic Framework: Boosting Electron − Hole Separation via Electron Trap States. *J. Am. Chem. Soc.* 2015, **137**, 13440–13443.

(13) Y. Lee, S. Kim, J. Kang and S. M. Cohen, Photocatalytic CO_2 Reduction by a Mixed Metal (Zr/Ti), Mixed Ligand Metal-Organic Framework under Visible Light Irradiation. *Chem. Commun.* 2015, **51**, 5735–5738.

(14) Y. Fu, D. Sun, Y. Chen, R. Huang, Z. Ding, X. Fu and Z. Li, An Amine-Functionalized Titanium Metal-Organic Framework Photocatalyst with Visible-Light-Induced Activity for CO_2 Reduction. *Angew. Chemie - Int. Ed.* 2012, **51** , 3364–3367.

(15) A. Chandran P., G. Radha, P. C. Meenu, S. Roy and H. Aggarwal, Post-Synthetic Exchange in a Zirconium Metal-Organic Framework for Efficient Photoreduction of CO_2 to Formate. *Mater. Adv.* 2024, **5**, 6936–6943.

(16) S.Karmakar, S. Barman, F. A. Rahimi, D. Rambabu, S. Nath, T. K. Maji, . Nat. Commun. Confining Charge-Transfer Complex in a Metal-Organic Framework for Photocatalytic CO_2 Reduction in Water, 2023, **14**, 4012.

(17) R. Reji, J. P. Vizuet, X. Yan, B. K. Behera, X. Zheng, M. Klenk, N. P. Weingartz, G. T. Kostopoulos, L. X. Chen, P. Zapol, N. Agarwal, E. Huerta, M. Keceli, S. Chaudhuri and K. D. Glusac, Photochemical CO_2 Reduction by a Postsynthetically Modified Zr- MOF. 2025, **64**, 18304–18315.

(18) D. Liu, T. Ouyang, R. Xiao, W. Liu and D. Zhong, Anchoring Co II Ions into a Thiol-Laced Metal – Organic Framework for Efficient Visible-Light-Driven Conversion of CO_2 into CO. 2019, **510275**, 2166–2170.

(19) B. Wang, W. Li, J. Liu, T. Gan, S. Gao, L. Li, T. Zhang, Y. Zhou, Metal-Modified Zr-MOFs with AIE Ligands for Boosting CO_2 Adsorption and Photoreduction. 2025, **2407154**, 1–9.

(20) L. Xia, W. Zhou, Y. Xu, Z. Xia, X. Wang, Q. Yang, G. Xie, S. Chen and S. Gao, Ferrocene-Boosting Zr-MOFs for Efficient Photocatalytic CO_2 Reduction : A Trade-off between Enhancing LMCT and Frustrating Lewis Acid. *Chem. Eng. J.* 2023, **451**, 138747.

(21) BIOVIA, Dassault Systèmes. *Materials Studio*, version 2019; Dassault Systèmes: San Diego, CA, **2019**.

(22) A. K. Rappe, C. J. Casewit, K. S. Colwell, W. A. Goddard and W. M. Skiff, UFF, a full periodic table force field for molecular mechanics and molecular dynamics simulations. *J. Am. Chem. Soc.*, 1992, **114**, 10024-10035.

(23) J. A. Pople, P. M. W. Gill and B. G. Johnson, Kohn—Sham density-functional theory within a finite basis set. *Chem Phys Lett*, 1992, **199**, 557–560.

(24) J. Moellmann and S. Grimme, DFT-D3 Study of Some Molecular Crystals. *The Journal of Physical Chemistry C*, 2014, **118**, 7615–7621.

(25) L. Goerigk, Chapter 6 - A Comprehensive Overview of the DFT-D3 London-Dispersion Correction. *Non-Covalent Interactions in Quantum Chemistry and Physics*, Elsevier, 2017, 195–219.

(26) J. Hafner, Ab-initio simulations of materials using VASP: Density-functional theory and beyond. *J Comput Chem*, 2008, **29**, 2044–2078.

(27) K. F. Garrity, J. W. Bennett, K. M. Rabe and D. Vanderbilt, Pseudopotentials for high-throughput DFT calculations. *Comput Mater Sci*, 2014, **81**, 446–452.
